# Supplementary material for: Development of a Potent Engineered Microbial Lipase for the Treatment of Exocrine Pancreatic Insufficiency
Source: Gastro Hep Adv. 2026 Apr 28;5(7):100984. doi: 10.1016/j.gastha.2026.100984 (PMC13241774; doi:10.1016/j.gastha.2026.100984)
Supplement: Supplementary Information [file mmc1.pdf]

Supplementary Information

Development of a potent, engineered microbial lipase for the treatment of exocrine pancreatic insufficiency

Christos S. Karamitros[1*], Chinping Chng[2], William Casey Hallows[2], Ravi Garcia[2], Kristen Skvorak[2], Adam Silverman[2], Nikki Kruse[2], Judy Viduya[2], Stephanie Galanie[2], Da Duan[2], Kerryn McCluskie[2], Bob Sato[2],John Watson[1], Alek Zajac[1], Carmine D'Urzo[1], Sharan Ghuman[1], Edward Edson[1], Ismail Aouadi[1], Chris Wynne[3], John Windsor[4], Gjalt Huisman[2], Bernard Cuenoud[1]

[1]Nestle Health Science, Lausanne, 1000, Switzerland

[2]Codexis Inc., Redwood City, California, USA

[3]New Zealand Clinical Research, Christchurch, New Zealand

[4]University of Auckland, Auckland, New Zealand

***Corresponding author**

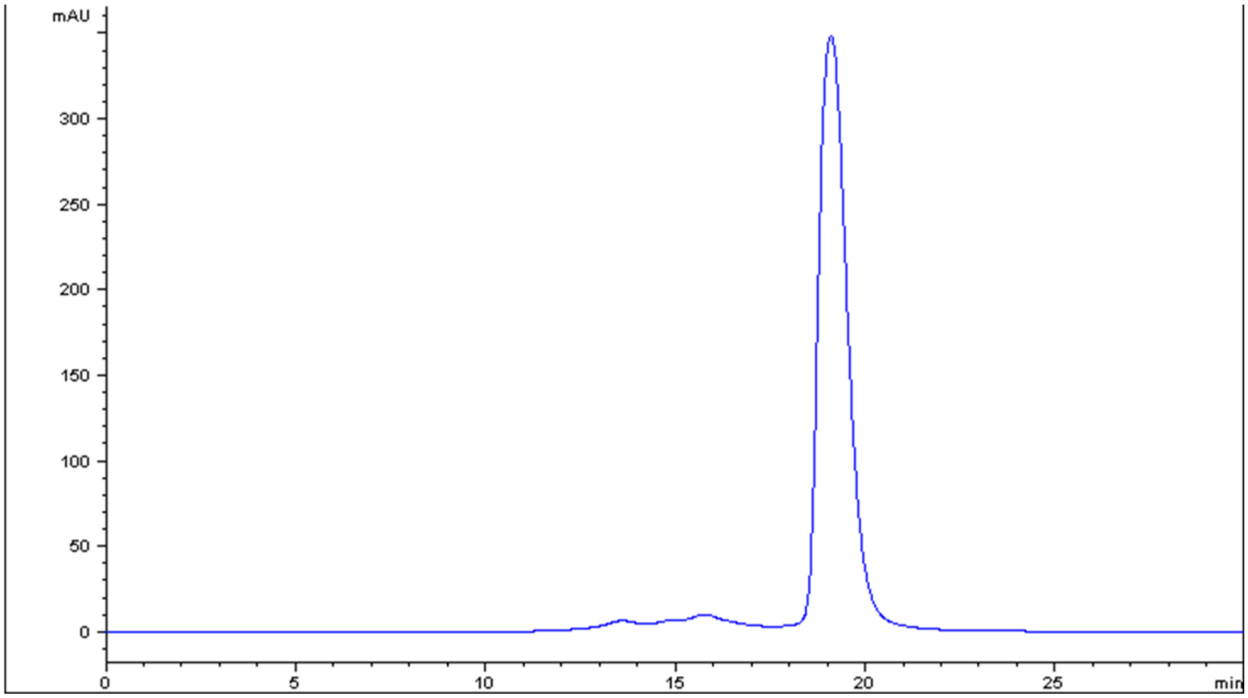

Peak	%Area A^{215}
HMW	9
NHS7108	91

Supplementary Figure 1. Analytical size-exclusion chromatogram (aSEC) of purified NHS7108. In the graph, Y- and X-axis represent the $Absorbance^{215}$ and the retention time (minutes) respectively. HMW: High-Molecular-Weight species.

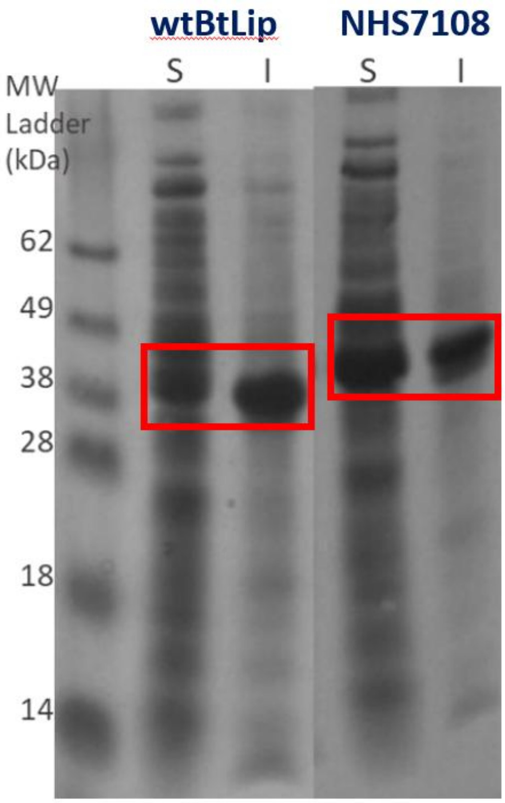

Supplementary Figure 2. SDS-PAGE of reconstituted enzyme powder of wtBtLip and NHS7108 expressed in *E. coli*. S and I indicate soluble and insoluble fractions whereas the red squares show the respective lipase bands (theoretical molecular weight: 43.6 kDa).

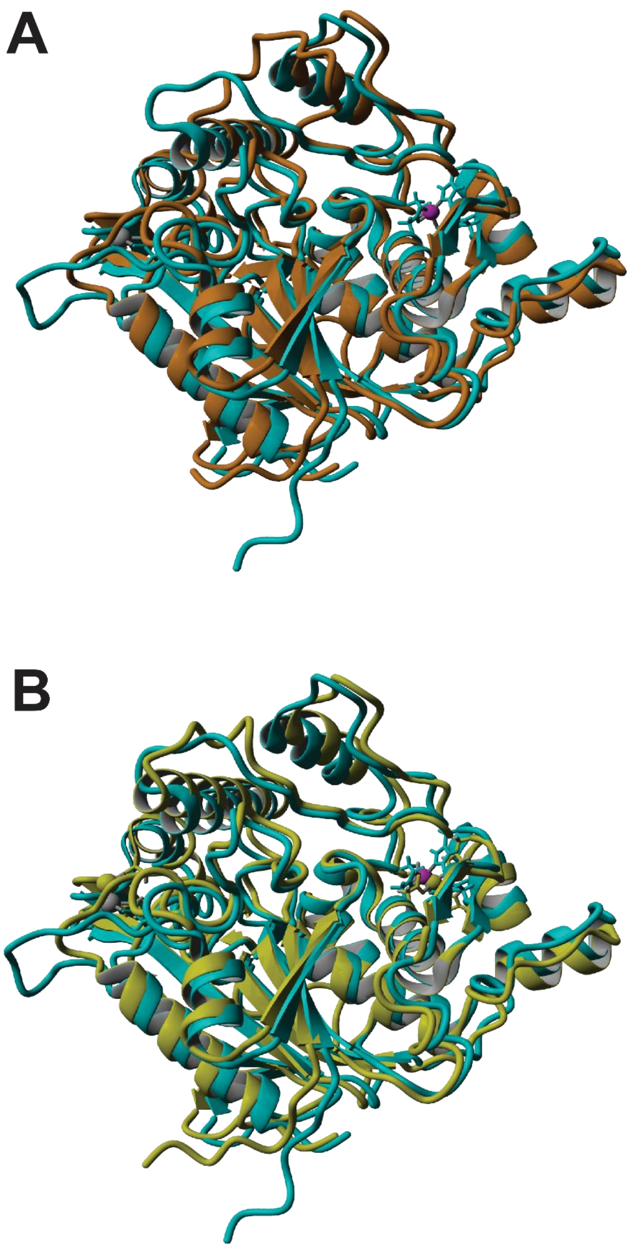

Supplementary Figure 3. NHS7108 homology model overlaid against structures of other microbial lipases which were experimentally determined by x-ray crystallography. (A) NHS7108 (cyan) vs T1 lipase (orange) from *G. zalihae* (PDB entry: 2DSN). (B) NHS7108 (cyan) against L1 lipase (yellow) from *B. stearothermophilus* (PDB entry: 1KU0) respectively. NHS7108 shares ~ 47% amino acid identity with each of the lipases whereas the RMSD of both structural alignments is ~5.3 Å. All the structural analysis in this study was done with the program YASARA Structure unless otherwise stated.

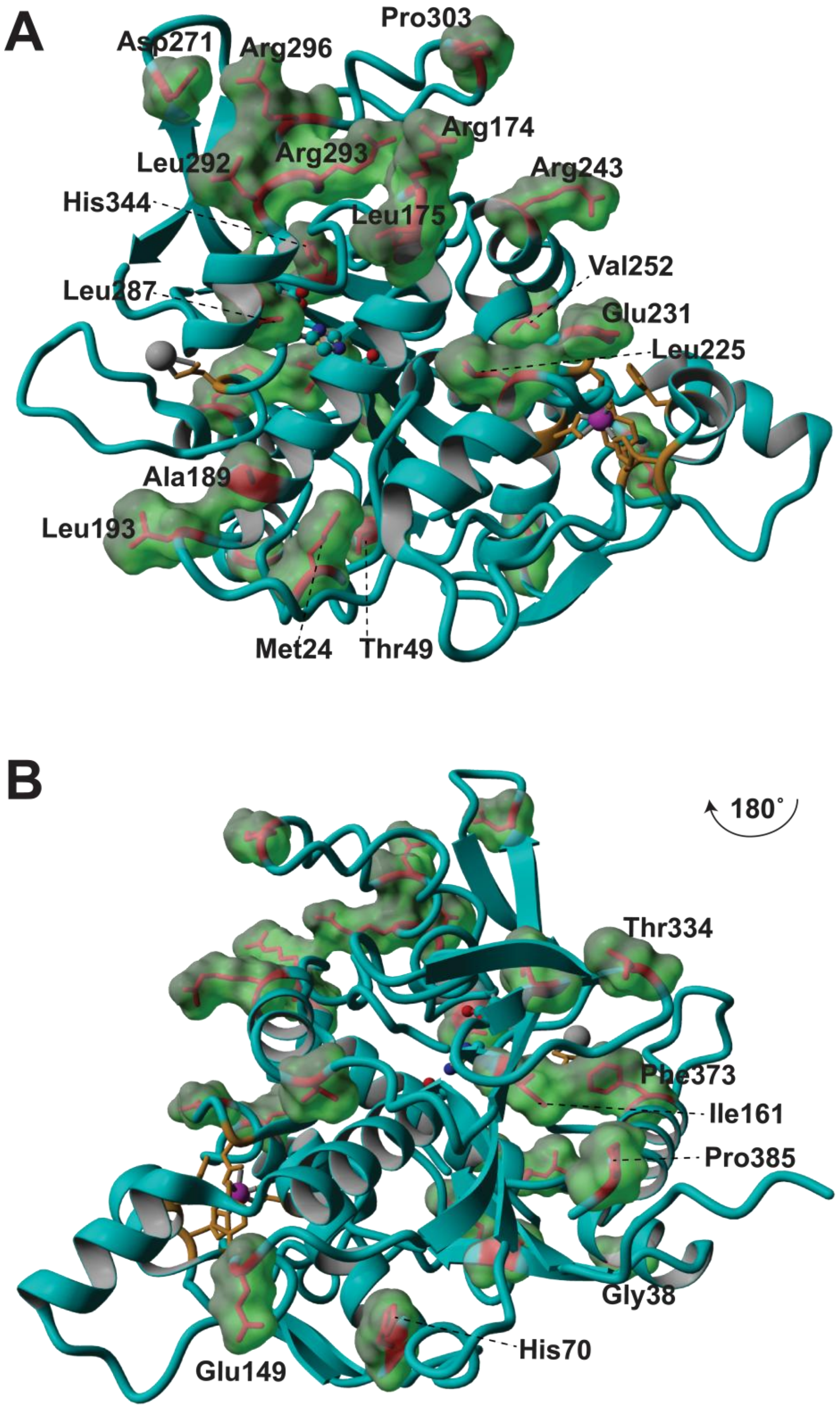

Supplementary Figure 4. (A) The modeled structure of NHS7108 displaying 24 amino acid substitutions, shown as red sticks and labeled in black. Residues interacting with Zn^{2+} (magenta sphere) and Ca^{2+} (grey sphere) metals are depicted in orange. (B) A 180º rotation of the structure is provided to enhance visualization of the amino acid substitutions at positions Gly38, His70, Glu149, Ile161, Thr334, Phe373, and Pro385.

Supplementary Table 1. Summary of the administration of various doses of NHS7108 and commercial PERT to PDL minipigs, along with the corresponding calculated %CFA values for each treatment group. Pre- and post-dose %CFA values were determined using data from the final count of animals per group as shown below, excluding minipigs with pre-dose %CFA above 60%. Data are represented as mean ± SEM. Δ is defined as ($\%CFA^{post\text{-}dose}$-$\%CFA^{pre\text{-}dose}$). (As outlined in the Methods section, purified NHS7108 was administered as lyophilized fermentation powder, while PERT was given in the form of enterically coated minitablets.

				**%CFA**			
**Treatment**	**Units/day (in 1000s)**	**Amount (g)**	**Final number of animals per group**	**Pre-surgery**	**Pre-dose**	**Post-dose**	**Δ**
NHS7108	33.7	0.055	2	93±1.5	40±4	65.5±2	25.5±6
	100	0.17	3		37.5±4	74±1	36.5±5
	301	0.5	3		42±6	81±1	39±7
PERT	80	1.4	4		50±7	72±3	22±10
	200	3.4	4		44±4	78±4	34±8
	400	6.8	4		44.5±4	84±2	39.5±6
