# Supplementary material for: Development of a Potent Engineered Microbial Lipase for the Treatment of Exocrine Pancreatic Insufficiency
Source: Gastro Hep Adv. 2026 Apr 28;5(7):100984. doi: 10.1016/j.gastha.2026.100984 (PMC13241774; doi:10.1016/j.gastha.2026.100984)
Supplement: Extended PDF [file mmc2.pdf]

Gastro Hep Advances 2026;5:100984

ORIGINAL RESEARCH—BASIC

Development of a Potent Engineered Microbial Lipase for the Treatment of Exocrine Pancreatic Insufficiency

Christos S. Karamitros,[1] Chinping Chng,[2] William Casey Hallows,[2] Ravi Garcia,[2] Kristen Skvorak,[2] Adam Silverman,[2] Nikki Kruse,[2] Judy Viduya,[2] Stephanie Galanie,[2] Da Duan,[2] Kerryn McCluskie,[2] Robert K. Sato,[2] John Watson,[1] Alek Zajac,[1] Carmine D'Urzo,[1] Sharan Ghuman,[1] Edward Edson,[1] Ismail Aouadi,[1] Chris Wynne,[3] John Windsor,[4] Gjalt Huisman,[2] and Bernard Cuenoud[1]

[1]*Nestle Health Science, Lausanne, Switzerland;* [2]*Codexis Inc, Redwood City, California;* [3]*New Zealand Clinical Research, Christchurch, New Zealand; and* [4]*Surgical and Translational Research Centre, Department of Surgery, Faculty of Medical and Health Sciences, University of Auckland, Auckland, New Zealand*

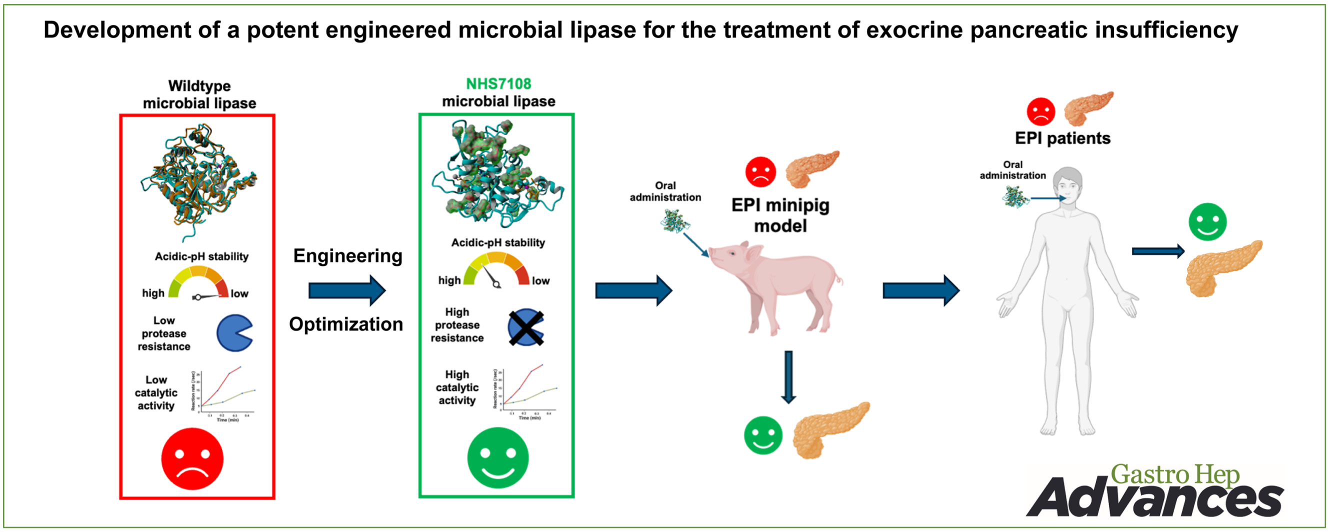

BACKGROUND AND AIMS: Exocrine pancreatic insufficiency (EPI) is a chronic condition associated with inadequate digestive enzyme secretion from the pancreas, causing impaired digestion, malabsorption, and malnutrition. EPI affects 10%–20% of the general population, and the standard of care includes pancreatic enzyme replacement therapy (PERT) that is exclusively derived from porcine pancreatic extracts. However, PERT is associated with several limitations, including suboptimal efficacy, which results in increased pill burden and high treatment costs. This work aimed to identify and engineer a potent microbial lipase that can function in the gastrointestinal tract without needing enteric coating and capable of hydrolyzing a wide range of dietary fats for the development of an alternative PERT. **METHODS:** State-of-the-art protein engineering and biochemical characterization techniques were applied to optimize and characterize a therapeutically relevant lipase. The lipase was evaluated in a pancreatic duct ligation minipig model, as well as in a clinical trial involving healthy subjects and EPI patients. **RESULTS:** The engineered lipase exhibited very high stability under simulated gastric and intestinal conditions while it can hydrolyze a wide range of dietary fats. Coefficient of fat absorption analysis demonstrated that the enzyme enabled pancreatic duct ligation minipigs to digest fat at levels comparable to porcine-derived PERT while using 10-fold lower dosing by mass of non–enteric-coated recombinant protein vs porcine PERT. Clinically, patients with EPI receiving orally administered lipase demonstrated a favorable safety profile and improvement of fat absorption as assessed by a $^{13}CO_2$-mixed triglyceride breath test in a proof-of-concept, integrated

Abbreviations used in this paper: **%CFA, percent coefficient of fat absorption; AE, adverse event; CF, cystic fibrosis; CFA, coefficient of fat absorption; EPI, exocrine pancreatic insufficiency; GI, gastrointestinal; HTP, high throughput; PD, pharmacodynamics; PDL, pancreatic duct ligation; PERT, pancreatic enzyme replacement therapy; PK, pharmacokinetics; TEAE, treatment-emergent adverse event; USP, United States Pharmacopeia; wtBtLip, wild-type *Bacillus thermoamylovorans* triacylglycerol lipase; YASARA, Yet Another Scientific Artificial Reality Application.**

Most current article

© 2026 Nestlé. Published by Elsevier Inc. on behalf of American Gastroenterological Association Institute. This is an open access article under the CC BY license (http://creativecommons.org/licenses/by/4.0/).
2772-5723
https://doi.org/10.1016/j.gastha.2026.100984

phase 1a-1b clinical trial. **CONCLUSION:** These results provide a foundation for the clinical development of a novel engineered recombinant lipase for treating EPI and warrant further evaluation in clinical trials.

Keywords: Exocrine Pancreatic Insufficiency; PERT; Engineered Lipase; Pancreatic Duct Ligation Minipig Model; Clinical Trial

Introduction

Exocrine pancreatic insufficiency (EPI) is a disorder that is characterized by the inability of the pancreas to produce and secrete adequate amounts of digestive enzymes (lipases, proteases, and amylase), resulting in impaired digestion and potentially malnutrition.[1] Many different conditions can cause irreversible EPI, but the most common ones include cystic fibrosis (CF) and chronic pancreatitis in children and adults, respectively.[2,3] The clinical manifestations of EPI predominantly include abdominal discomfort, steatorrhea, poor fat, and fat-soluble vitamin absorption, while the diagnosis can be a lengthy, multistep process.[4]

Pancreatic enzyme replacement therapy (PERT) is considered the gold standard for treating EPI. Its aim is to provide active digestive enzymes to the duodenum and proximal jejunum simultaneously with food intake, thereby improving nutrient absorption. Currently, the majority of the FDA-approved PERT products are based on pancreatin, which is a crude mixture derived from multiple fractionation steps of freeze-dried porcine pancreatic glands.[5] Pancreatin and its enzyme-enriched version pancrelipase are often formulated in coated microbeads, microspheres, or minitabs to resist acid-induced deactivation as well as proteolytic degradation by pepsin in gastric fluids, with pancreatic lipase being the least stable species.[6,7] In the duodenum's nearly neutral pH ($\geq$6.0), the coating dissolves, releasing active enzymes to aid food digestion.

However, while PERTs are the primary treatment for EPI patients, they have notable limitations. First, because pancrelipase-based PERTs are impure and not highly enriched for and concentrated with digestive enzymes, they contain low levels of pancreatic lipase, requiring multiple capsules per meal to reach the enzyme units needed for substantial fat digestion. Second, research indicates that EPI patients experience prolonged acidic intraduodenal pH ($\leq$5) due to reduced pancreatic bicarbonate secretion, which is essential for neutralizing stomach acid. Extended acidic conditions in the duodenum slow down the dissolution of the enteric coating, which delays enzyme release, and liberated pancreatic lipase may be deactivated by the low pH environment. Third, it has been postulated that there is inadequate mixing of the enzymes with the food chyme during the delivery of the enteric-coated drug product in the intestine, and thus a significant proportion of nutrients are not exposed to digestive enzymes.[8–10] Collectively, these limitations mentioned above lead to a high "pill burden" for EPI patients, who need to take many pills daily to aid digestion.[11–13]

To address the shortcomings of existing PERTs, various groups have focused on substituting pancreatic lipase with microbial counterparts.[14] Unlike the pancreatic lipase which requires colipase as cofactor and is neither stable nor active at low pH, microbial lipases appear to be more attractive candidates due to their higher stability, independence from colipase, and established industrial-scale production. These efforts have progressed through various stages of preclinical and clinical development[15–19] but, as yet, none have received FDA approval. Liprotamase (or Sollpura), a triple-combination of cross-linked bacterial lipase, protease, and amylase advanced the furthest in clinical trials but was denied FDA approval for the treatment of CF-related digestive problems in 2019 due to insufficient efficacy in a phase-3 clinical trial.[20] Anagram Therapeutics recently announced encouraging findings from their phase-1 clinical trial, in which a triple recombinant combination of engineered lipase, protease, and amylase was evaluated for safety and dose response in patients with CF.[21]

In this study, we present the preclinical and clinical development of a novel, engineered microbial lipase for the treatment of EPI. New triacylglycerol lipase variants with the desirable phenotypic traits were identified after multiple rounds of optimization by applying CodeEvolver protein engineering (Codexis Inc) using the wild-type *Bacillus thermoamylovorans* triacylglycerol lipase (wtBtLip) as starting template. The lead molecule, NHS7108 (formerly known as CDX7108), exhibited high gastric and intestinal protease stability in addition to resistance to acidic pH and high temperatures while retaining its lipase activity in vitro. Furthermore, in in vivo tests in a pancreatic duct ligation (PDL) minipig model of EPI, NHS7108 restored the percent coefficient of fat absorption (%CFA) to healthy levels with 10-fold lower dosing by mass than commercial PERTs. In a proof-of-concept crossover, placebo-controlled phase-1 clinical study, in which EPI patients were enrolled, administration of NHS7108 improved the lipid absorption of the treated group by 72% relative to the placebo as measured by cumulative $^{13}CO_2$-mixed triglycerides (MTG) breath test. Taken together, our preclinical and early clinical findings indicate that NHS7108 constitutes a promising candidate for an alternative PERT.

Materials and Methods

Directed Evolution Experiments

Briefly, for directed evolution and engineering experiments,[22] a DNA sequence encoding wtBtLip was codon-optimized for recombinant expression in *Escherichia coli* and cloned into an expression plasmid under the control of the isopropyl-β-D-thiogalactopyranoside–inducible *lac* promoter. The mutant libraries were designed and

screened by employing a directed evolution protein engineering platform technology (CodeEvolver) consisting of several steps including (1) generation of sequence diversity by employing PCR methods, (2) high-throughput (HTP) phenotype screening of the variants upon expression in the *E coli* host, (3) next-generation sequencing coupled to data analysis, and (4) selection of beneficial mutations for next evolution rounds as needed.[23] The *Bacillus thermocatenulatus* lipase variants were screened and selected based on their ability to retain their catalytic activity upon exposure to elevated temperature, low pH, pepsin, trypsin, and chymotrypsin challenge experiments as described in the following section.

Biochemical Characterization of NHS7108

For pH stability analysis, 50 μL of lipase variants from HTP lysates were mixed with either 50 μL of 50 mM sodium citrate (pH: 2.0–5.0) or 50 μL of 50 mM sodium phosphate (pH: 5.0–8.0) in a 96-well round bottom plate. The plate was sealed and incubated for 2 hours at 37 °C with agitation at 300 rpm. After the incubation period, 10 μL of the challenged mixture was tested for catalytic activity. The same setup and buffers were used for stability tests against pepsin, trypsin, and chymotrypsin.[22] The residual catalytic activity of lipase was measured by its ability to hydrolyze triolein by applying the RapidFire mass spectrometry (Agilent Technologies, Santa Clara, CA) method as follows: ~80 μL of 100 mM sodium phosphate pH 7.0 and 2.5 μL triolein (25 mM final concentration in reaction) were mixed with 10 or 20 μL of lysate or challenged mixture (as described above) in a 96-well polypropylene deep-well plate, sealed and incubated for 1 hour at 37 °C with agitation at 300 rpm. In some assays, 5 mM sodium taurocholate to simulate the presence of bile salts was added to the reaction. Reactions were quenched with 600 μL acetonitrile 2-propanol:isopropyl alcohol (IPA;1:3), clarified with centrifugation at 4000 rpm for 5 minutes and diluted 10 μL into 190 μL 50:50 IPA:methanol, mixed then diluted once again by adding 10 μL into 190 μL of 50:25:25 H2O:IPA:methanol. Lipase activity was measured at pH = 7.0, unless stated otherwise. The same process was followed for the characterization of NHS7108 and wtBtLip solid formulations which were prepared by a laboratory-scale fermentation process (as described in section below). Before mixing NHS7108 and wtBtLip with the desirable buffer for each experiment, the purified lyophilized powders were initially reconstituted in water at a stock concentration of 2 mg/mL. For pH activity profiling of pancrelipase, enterically coated microspheres were removed from the capsule and were dissolved in 40 mL phosphate-buffered saline under shaking conditions until fully solubilized (~30 minutes). The reactions were performed in different pH values for 1 hour as described above and were analyzed by performing the RapidFire mass spectrometry assay. The simulated ingestion experiment was conducted using NHS7108 shake-flask lyophilized powder, pancreatin and pancrelipase (intact enterically coated microspheres). The enzyme preparations were mixed with pepsin in sodium citrate buffer, pH 2.5 as described above, incubated for 1 hour at 37 °C with agitation at 300 rpm. After the 1 hour preincubation, each of the pepsin-treated enzyme preparation was added to olive oil substrate in the pH-stat in the presence of 2 mg/mL of trypsin and 2 mg/mL chymotrypsin. The pH-stat was run with the modification that the reaction was performed at pH 6.5 and for 3 hours. The amount of 0.1N NaOH added was monitored to determine the activity.

NHS7108 Production by Shake-Flask and Fermentation

Shake-flask crude lyophilized powder of wtBtLip and NHS7108 was produced after expressing the enzymes in *E coli*. Briefly, strains were grown in 250-mL shake-flask scale and induced for expression with isopropyl-β-D-thiogalactopyranoside. In the 250 mL scale, the cell pellet was resuspended in 30 mL phosphate-buffered saline, pH 7, subsequently lysed by a single pass through a microfluidizer, and clarified by centrifugation. Following clarification, supernatant was lyophilized, and the enzyme powder was stored at −20 °C until use. Conversely, purified solid NHS7108 was produced by following a typical, fully controlled (media, pH, and oxygen) fermentation process consisting of the following 2 phases: growth and expression. At the end of the fermentation, cells were harvested by bucket centrifugation, and the pellets were stored at −80 °C until further use. Cell pellets were thawed overnight at 4 °C, combined, and resuspended in Processing Buffer (50 mM Tris, 137 mM NaCl, 2.7 mM KCl, pH 9.0) to a final volume equal to the fermentation harvest volume. The cell resuspension was lysed by rapid pressure change using a homogenizer fitted with appropriate disruption valve. Whole lysates were flocculated and clarified by centrifugation. The product was salted out by the addition of sodium sulfate to the clarified supernatant to a final concentration of 0.8 M and collected by centrifugation. Supernatant was discarded, and the resulting pellet was resolubilized in Processing Buffer and sterilized by passage through a PES 0.2 μm pore size membrane filter (Sartopore 2 XLG). The sterile filtrate was lyophilized to obtain a free-flowing, off-white, powder which was stored at −20 °C until use.

pH-Stat Activity Assay

NHS7108 enzyme fermentation powder was constituted in sterile water to provide a 10 mg/mL stock solution, and the specific activity was determined by a protocol adapted from the United States Pharmacopeia (USP) pancreatin lipase assay (USP and National Formulary [(USP42-NF 37), 2016)]). One USP unit of lipase activity is contained in the amount of pancreatic lipase that liberates 1.0 μEq of acid per minute at pH 9.0 °C and 37 °C under conditions of the assay. The purity of lipase powder was determined by size

exclusion chromatography and the protein content in lipase powder was determined with bicinchoninic acid assay. Finally, the enzyme content of NHS7108 powder was determined by multiplying the enzyme purity and protein content.

In Silico Molecular Modeling

Homology modeling of wtBtLip and NHS7108 was done with the Yet Another Scientific Artificial Reality Application (YASARA) Structure program using the macro "hm_build" as described in other reports.[24,25] Briefly, the program screened the Uniprot database (https://www.uniprot.org/) using the target sequence of the enzymes to identify related protein sequences and subsequently, this profile was used to search Protein Data Bank (PDB) for potential modeling templates. Finally, the identified templates were ranked based on alignment and structural quality scores as described previously.[26] The resulting structure based on the identified wtBtLip and NHS7108 models showing the highest score was the T1 lipase from *Geobacillus zalihae* (PDB code: 2DSN). The final structural models as predicted by YASARA were independently evaluated by submitting them to the MolProbity server (http://molprobity.biochem.duke.edu/) and their quality was assessed as "good". Finally, the models were subjected to further analysis by YASARA Structure.

In Vivo Dose Response With NHS7108 in a PDL Minipig Model

All experiments involving minipigs were conducted at AltaScience (Auxvasse, MO). Throughout the studies, all animals were assessed and monitored by veterinary personnel, with every procedure being endorsed and approved by the local animal ethics committee. Twenty-four *Sus scrofa* female animals were subjected to PDL surgery.[27] In the first NHS7108 study, animals were up to 7 months old and weighed 12–20 kg before dosing, while in the second PERT study, minipigs were around 1.6 years old and weighed 30–42 kg. Based on the weight of each animal, approximately 400–600 (±5) grams of S-9 Swine Feed was measured and blended 10:1 with 40–60 (±1) grams of olive oil (Feeding mix #1). For the NHS7108 treatment, the correct amount of enzyme powder was measured and thoroughly blended with unsweetened apple sauce (Feeding mix #2). The 2 feeding mixtures were combined, and the total weight was recorded before presenting it to the minipig for consumption. The final feeding mixture with PERT, consisting of enterically coated minitablets, was prepared using the same procedure. Every animal was weighed during both the acclimation and dosing periods. After the surgery, some animals received daily doses of maintenance Viokase to aid their digestion until all animals had completed the surgery. During acclimation the pigs were randomized into different groups of 4 pigs followed by 10 days of dosing with either NHS7108 or commercial PERT. Acclimation lasted 11 and 14 days for NHS7108 and PERT, respectively. Fecal samples were collected over the final 3 acclimation and dosing days, beginning after feeding on the initial day and concluding before feeding the following day. All fecal material produced over the 24-hour period between 2 feedings was collected periodically and total weight determined. Fecal samples were then analyzed for fat content using the modified Van de Kamer method.[28]

Phase 1: Clinical Evaluation of NHS7108

The safety, pharmacokinetics (PK), and proof-of-concept efficacy of a liquid oral solution of NHS7108 (25 mM Tris, 150 mM NaCl, 10% w/w sucrose, pH 8.5 with NHS7108 at 30 mg/mL) were investigated in a phase 1a-1b, integrated, 3-part (parts A, B, and C) clinical study (NCT05082051) conducted in 4 sites in Australia and New Zealand. Participants provided written informed consent prior to the beginning of the study. The study was approved by the respective Ethics Committee in each country and was conducted in accordance with the Declaration of Helsinki. Parts A and B were randomized, double-blind, placebo-controlled dose escalation groups to investigate safety and PK after single and multiple oral dose administration in healthy adult subjects. Part C was a randomized, double-blind, placebo-controlled, single-dose, 2-way crossover part to investigate safety and pharmacodynamics (PD) of NHS7108 in subjects with severe EPI. The ^{13}C-MTG Pancreo-Lip breath test was used to assess PD.[29] Dose escalation and transition across study parts occurred in a sequential fashion, contingent on review of safety, tolerability, and available PK data of the previous dose level.

For parts A and B, healthy male and female subjects between the ages of 18 and 55 years, with a body mass index between 18.0 and 30.0 kg/m^2 were recruited. Part C recruited male and female subjects between 18 and 75 years, with a body mass index between 18.0 and $\leq$35.0 kg/m^2 who had severe EPI from total or partial pancreatectomy or chronic pancreatitis and were clinically well controlled under the regular use of PERT. Severe EPI was defined as fecal pancreatic elastase-1 <100mcg/g and 150_{min} %$^{13}CO_2$ excretion rate $\leq$4.4% dose/h at the Pancreo-Lip breath test. The main reasons for exclusion were allergies or adverse reaction to NHS7108 components, ongoing or recent investigational treatment, or medical conditions entailing a safety risk or potentially interfering with the study procedures. Additional exclusion criteria for part C only were celiac disease, food intolerance or allergy, any medical conditions affecting the gastrointestinal (GI) function, and medications affecting gastric pH, GI motility or fat absorption (histamine blockers and proton pump inhibitors were allowed).

The study end points included safety, PK (parts A and B only), and PD (part C only) assessments. More specifically, the safety end points included adverse events (AEs) and changes in clinical laboratory tests, vital signs, 12-lead ECG, and

physical examination. The PK end points were assessed as follows: for part A AUC0-t, AUC0-inf, C_{max}, T_{max}, t1/2, and λz and for part B $AUC_{0-\tau}$, AUC_{0-t}, $C_{ss,max}$, 6 C_{τ} (predose) at day 1 and day 6, T_{max}, and t1/2. The PD end points included ^{13}C-MTG Pancreo-Lip breath test fat absorption parameters,[30] including percentage $^{13}CO_2$ excretion rate over baseline (% dose/h); 150_{min} percentage $^{13}CO_2$ excretion rate (% dose/h); and 240_{min} $^{13}CO_2$ cumulative excretion (% dose).

Considering the exploratory nature of the study, the sample size was not based on formal statistical considerations. Any statistical testing was exploratory and descriptive. There were 3 planned analyses as follows: (1) a first analysis at completion of part B; (2) an interim analysis at completion of the first 5 subjects in part C; and (3) a final analysis at the completion of part C completed the end of study/early termination visit.

Each NHS7108 dose was administered orally in a 30 mL solution together with the meals, that is, breakfast in parts A and C and breakfast, lunch, afternoon snack and dinner in part B. In Part C, following a 3-day washout from the subjects' usual PERT (inclusive of an overnight fast of at least 10 h), NHS7108 was administered orally together with a standardized breakfast containing the ^{13}C-MTG substrate for the Pancreo-Lip breath test. The subject's breath was collected over the following 4 hours at 30-minute intervals.

Results

Identification and Engineering of a Potent Microbial Lipase for High Stability in the GI Tract

Following evaluation of several wild-type microbial lipases for their advantageous biochemical properties such as recombinant expression, thermodynamic stability, activity as a function of pH and the capability to hydrolyze various dietary fats, a triacylglycerol lipase from the thermophilic bacterium *B thermoamylovorans* (wtBtLip) was selected as the starting point for protein engineering.

WtBtLip was recombinantly expressed in *E coli* following either fed-batch fermentation or shake flask protocols followed by fractionation and lyophilization as described in the Methods section. The enzyme is monomeric as evidenced by analytical size-exclusion chromatography (Supplementary Figure 1) and consists of 386 amino acid residues (mature sequence without the peptide leader) migrating at ∼43.6 kDa on sodium dodecyl sulfate-polyacrylamide gel electrophoresis (Supplementary Figure 2). Sequence alignment analysis showed that wtBtLip is ∼47% identical to other well-characterized bacterial thermoalkalophilic lipases from *G zalihae* T1,[31] *Bacillus stearothermophilus* L1[32] and *Geobacillus stearothermophilus* T6[33] (Figure 1B). Like those thermoalkalophilic lipases, wtBtLip harbors the highly conserved catalytic triad *Ser-Asp-His* (at positions 112, 314, and 353, respectively; numbering based on the sequence of wtBtLip) as well as the conserved active site motif *Gly-X-Ser-X-Gly* (where *Ser* is the catalytic Ser112) (Figure 1B), classifying it as member of the I.5 family of lipases.[34] Of note, wtBtLip has 2 putative metal-binding sites for Zn^{2+} and Ca^{2+} based on the presence of metal-binding residues that superpose well with those from characterized homologs including L1 lipase from *B stearothermophilus* (PDB code: 1KU0) and T1 lipase from *G zalihae* (PDB code: 2DSN), respectively.[31,32] Importantly, a prior study[35] showed that lipase from *B thermoamylovorans* NB501 strain which shares 98% identity with wtBtLip is a very promiscuous catalyst that preferentially hydrolyzes triglycerides at positions 1,3, thereby strongly indicating that wtBtLip is a common sn1,3-specific lipase (Figure 1A).

Aiming at the development of a stable and orally compatible lipase, capable of hydrolyzing a wide range of dietary fats in the GI tract, wtBtLip was subjected to iterative rounds of directed evolution using 3 main selection pressures including (1) resistance against GI proteases (pepsin, trypsin, and chymotrypsin), (2) high stability at low gastric pH, and (3) increased catalytic activity until no further improvement could be achieved under the screening conditions.[22] Employing Codexis' proprietary protein engineering technology (CodeEvolver)[36,37] and screening *B thermocatenulatus* lipase variants under simulated gastric and intestinal conditions we identified NHS7108 after 7 rounds of directed evolution and introduction of 24 mutations relative to wtBtLip (Figure 1C).[22]

To gain additional insights on the molecule's structural conformation and the position of the acquired mutations a homology model for NHS7108 was constructed using the program YASARA Structure as described in Methods and other reports.[24,25,38] Indeed, the overall modeled structure of NHS7108 is very similar to that of T1 and L1 lipases from *G zalihae* and *B stearothermophilus*, respectively, harboring 2 putative metal-binding sites (Figure 2A, Supplementary Figure 3), while NHS7108's catalytic site (Ser112, Asp314 and His353) resides at a hydrophobic core in the center of the molecule (Figure 2B). NHS7108's 24 amino acid substitutions are predominantly distributed around the surface of the molecule and are located on loops far away from the active site and the metal binding domains (Figure 2C, Supplementary Figure 4).

Biochemical Characterization of NHS7108 in Simulated GI Conditions and Activity Against Dietary Fats

To evaluate the biochemical properties of NHS7108 obtained through directed evolution, we conducted an extensive biochemical characterization campaign and compared NHS7108 with wtBtLip, pancrelipase, and pancreatin. Triolein was chosen as a representative substrate for the characterization of NHS7108. As shown in Figure 3A, NHS7108 displays a broader pH stability profile as compared to wtBtLip after 2 hours of preincubation at the respective pH value in the range of 2.0–8.0, followed by activity measurement at pH 7.0. Remarkably, NHS7108 retains nearly 100% of

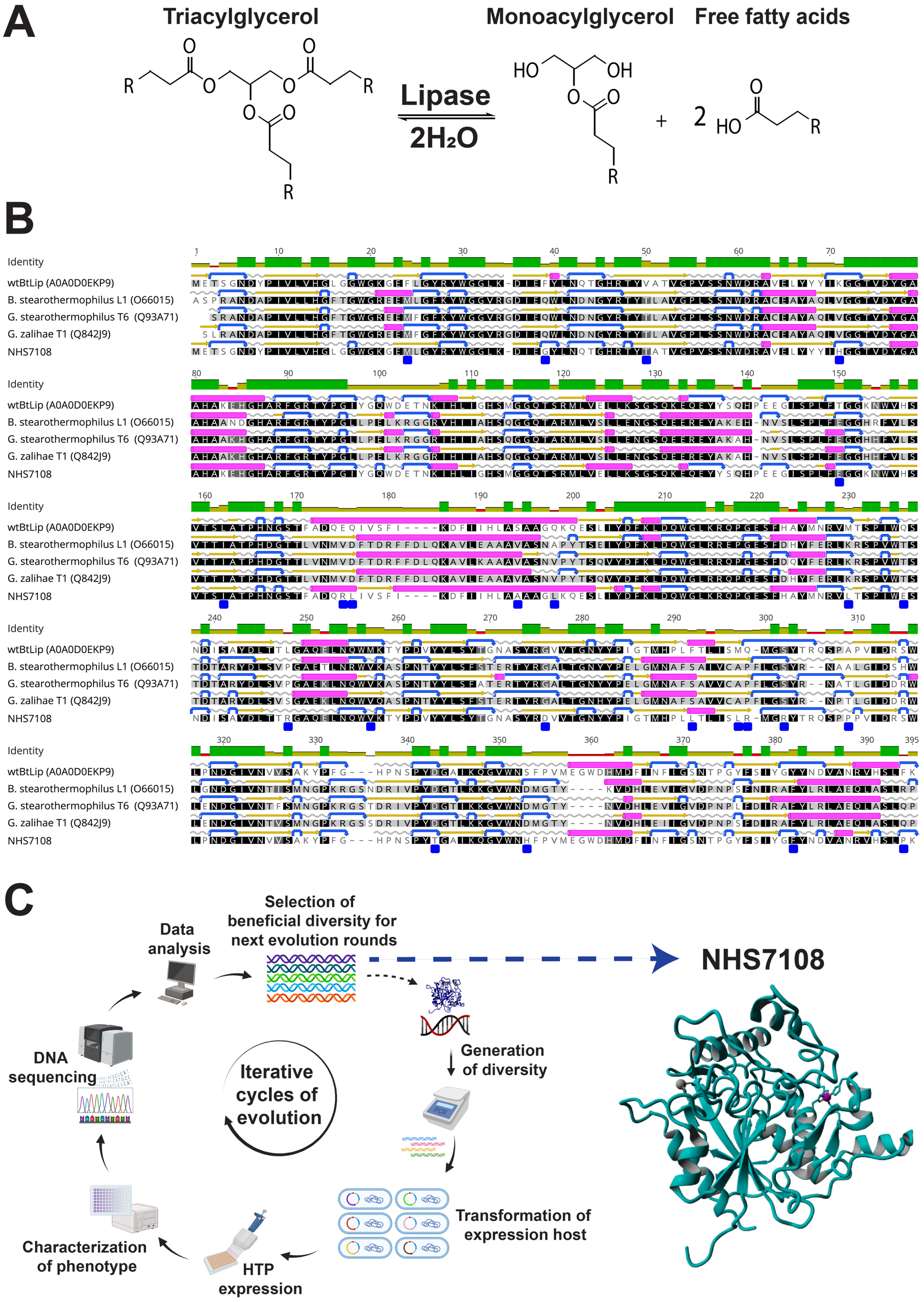
A
Triacylglycerol
Monoacylglycerol
Free fatty acids
Lipase
2H2O
+
2
B
Identity
wtBtLip (A0A0D0EKP9)
B. stearothermophilus L1 (O66015)
G. stearothermophilus T6 (Q93A71)
G. zalihae T1 (Q842J9)
NHS7108
C
Data analysis
Selection of beneficial diversity for next evolution rounds
NHS7108
DNA sequencing
Iterative cycles of evolution
Generation of diversity
Transformation of expression host
Characterization of phenotype
HTP expression

its activity even when it was incubated at pH as low as 2.0 whereas wtBtLip exhibits ~20% retained activity upon incubation at pH 4.0 and has no detectable activity following incubation at pH 2.0 and 3.0. Similarly, when NHS7108 was mixed with 2 mg/mL porcine pepsin at pH 2.5, the enzyme retained almost 100% of its initial activity after 5 hours of incubation at 37 °C (Figure 3B) in contrast to its wild-type counterpart that was completely deactivated within 30 minutes of incubation. In the presence of porcine intestinal proteases trypsin and chymotrypsin (2 mg/mL each), the residual activity of NHS7108 appeared to be 100% relative to the initial measurements across the 5 hours of incubation period. In contrast, wtBtLIP displayed a gradual, time-dependent decrease in activity indicative of proteolytic degradation (Figure 3C).

In addition to the data obtained from experiments using HTP lysates, we evaluated the stability and catalytic activity of purified NHS7108, which was prepared as lyophilized solid formulation following a fermentation process. NHS7108 displayed a broader activity profile in the pH range of 4.0–9.0 relative to pancrelipase (5.0–9.0) (Figure 3D). More specifically, NHS7108 showed activity >80% from pH 5.0–7.0 whereas the same activity for pancrelipase was observed at a narrower range (6.0–7.0). The higher pH activity of NHS7108 could be attributed either to a catalytic turnover increase or to a pH stability improvement or a combination of both. Notably, purified NHS7108 exhibited a very good stability profile when mixed and incubated with GI proteases. That is, after 2 hours of incubation with 1.5 mg/mL pepsin at pH 2.5, NHS7108's catalytic activity was not negatively affected, while in the presence of trypsin and chymotrypsin (1.5 mg/mL each), the lipase retained >80% of its initial activity at pH 7.0 (Figure 3E). Taken together, these data are in agreement with the proteolytic stability profile of the HTP lysates (Figure 3A–D). Finally, NHS7108 outperformed USP pancreatin and pancrelipase when all preparations were tested in a simulated ingestion experiment (consisting of an initial incubation in simulated gastric fluids followed by continuous catalytic activity determination in the presence of intestinal proteases employing the pH-stat method) (Figure 3F). Overall, our data indicate that NHS7108's enhanced stability and catalytic activity in simulated GI environments make it a promising candidate for developing a better PERT, especially due to its ability to act on diverse dietary fats (Figure 4).

NHS7108 Significantly Improves Fat Absorption in a PDL Minipig Model

Following the promising in vitro performance of NHS7108 in simulated GI conditions, its ability to hydrolyze physiologically relevant dietary fats and improve the digestion process, the enzyme was tested in a PDL minipig model.[27] Surgical PDL leads to the development of EPI in the minipig and represents a gold-standard preclinical animal model for testing and benchmarking PERTs.[27] A total of 24 female minipig animals underwent (PDL) surgery and were enrolled into 2 dose-response studies (12 animals each) during which they were treated with either NHS7108 or commercial PERT as control. In each study, the 12 animals were randomly divided into 3 groups of 4 pigs and each group received a predetermined dose of either NHS7108 or PERT (Supplementary Table) as illustrated in Figure 5A and described in the Methods section. The total fecal fat content of each animal from both the acclimation and dosing periods was assessed and based on the total consumed fat, the coefficient of fat absorption (CFA) was calculated. Of note, in some cases the final number of animals per group was ultimately reduced to either 2 or 3 due to likely reversion of the PDL surgery as evidenced by the calculated CFA during the acclimation phase (Supplementary Table).

Before surgery, the healthy animals from both studies showed a CFA of ~93 ± 1.5% (Figure 5B and C), whereas postsurgery, at the end of the acclimation phase, the predose CFA of the EPI animals was 40 ± 3% and 45 ± 4% for the NHS7108 (Figure 5B) and PERT (Figure 5C) studies, respectively. Administration of 3 NHS7108 dose levels ([low, medium, and high]; approximately 34K (0.055g of enzyme), 100K (0.17g), and 301K units (0.5g), respectively) led to progressively increased %CFA values of 65%, 74%, and 81%. These differences are statistically significant when compared to the predose %CFA value of 40% (Figure 5B). Corresponding ΔCFA values are detailed in Supplementary Table. In the control study, using porcine-based PERT also improved the animals' %CFA compared to predose phase. However, a higher dosing range (80K–400K; 1.4–6.8 g pancrelipase) was needed to attain %CFA comparable to the NHS7108-treated animals (Figure 5C, Supplementary Table). Notably, given the specific activity of NHS7108 in this study (~550–600 U/mg as

Figure 1. Identification and development of NHS7108. (A) Primary hydrolytic reaction catalyzed by wtBtLip and NHS7108 (sn1,3-specific) based on their sequence homology with other characterized microbial lipases. (B) Primary amino acid sequence alignment of wtBtLip, L1 lipase from *B. stearothermophilus*, T6 lipase from *G. stearothermophilus*, T1 lipase from *G zalihae*, and NHS7108. The Uniprot accession number for each enzyme species is shown in parenthesis. The level of identity among the 5 sequences is shown on the top row as green color gradient. α-helices, β-sheets, turns, and coils are represented as magenta bars, gold arrows, blue arrows, and gray lines, respectively. The positions of the 24 amino acid substitutions of NHS7108 are denoted as blue squares below the NHS7108 sequence. The sequence alignment was performed by Geneious Prime. (C) Directed evolution workflow employing Codexis' CodeEvolver proprietary technology that led to the identification of NHS7108.

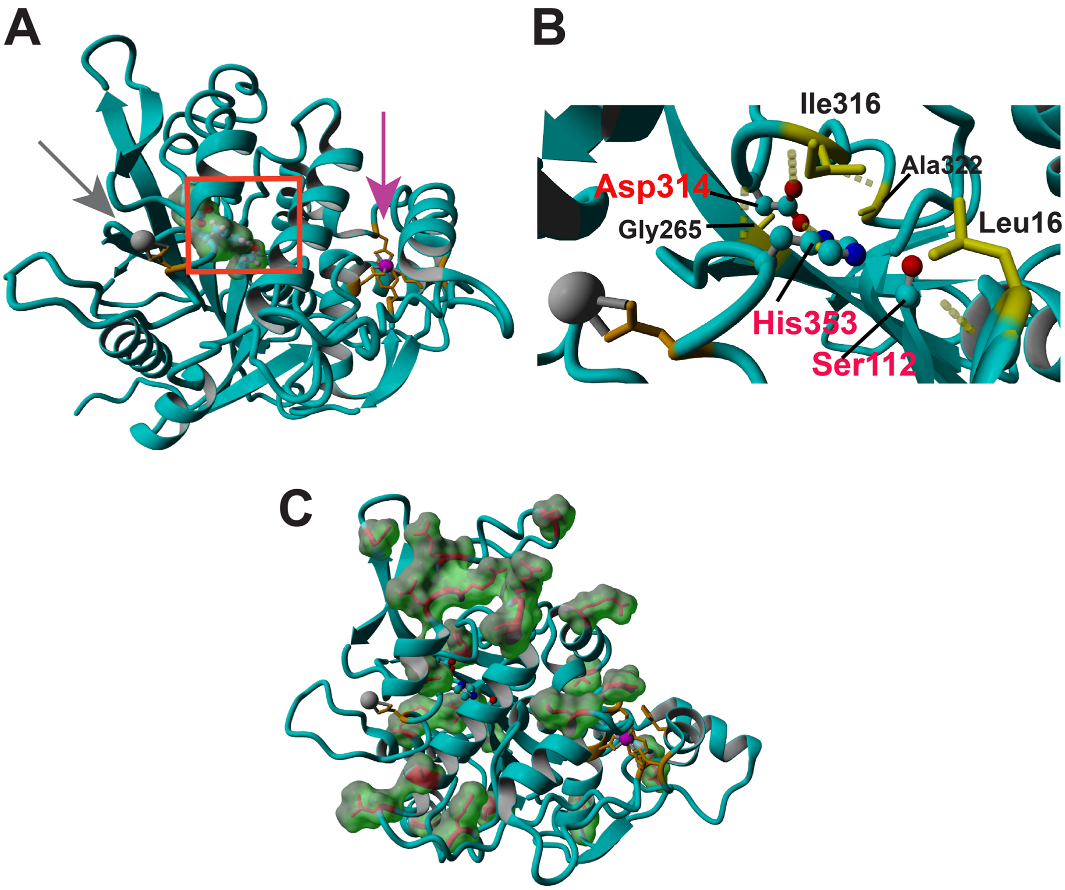

Figure 2. Homology modeling of NHS7108. (A) Structural representation model of NHS7108. The catalytic residues Ser112-Asp314-His353 are shown as spheres at the center of the molecule as defined by the red square. The molecular surface of the catalytic residues is also shown. The 2 putative metal binding sites for Ca^{2+} and Zn^{2+} are denoted with gray and magenta arrows, respectively, and metal-interacting residues are shown as orange sticks. (B) Zoom-in snapshot on the active site of NHS7108 showing the 3 key catalytic residues Ser112-Asp314-His353 as spheres in red fonts and neighboring residues (Leu16-Gly265-Ile316-Ala322) as yellow sticks and in black fonts. Hydrogen bonds between the catalytic and neighboring residues are shown as yellow dashed lines. (C) Mapped mutations on NHS7108's structural model. All 24 amino acid substitutions are shown as red sticks along with their molecule surface as green cloud like the other panels. In panels A and B the active site residues are color-coded by atom; nitrogen is blue, and oxygen is red.

calculated based on the pH-stat method) and the required quantities to achieve the desired dosage (Supplementary Table), this engineered lipase can restore %CFA to nearly healthy levels with ∼10-fold less dosing by mass compared to traditional PERT (highest dose of 301K units of NHS7108 corresponding to 0.5 g yielded 81% %CFA as compared to ∼6.8 g and 400 K units of PERT for 84% %CFA). Our PDL minipig data collectively suggest that NHS7108 functions effectively in vivo without requiring enteric coating or other similar protective formulations and uses markedly less active pharmaceutical ingredient compared to conventional PERTs, positioning it as a promising candidate for nonporcine recombinant PERT development.

NHS7108 Improves Lipid Absorption in EPI Patients

For the clinical assessment of NHS7108, a liquid drug product formulation was specifically developed and optimized, comprising tris(hydroxymethyl)aminomethane, sodium chloride, sucrose, and water at pH 8.5. Under these conditions, the enzyme was concentrated to 30 mg/mL and was frozen at temperatures ≤−60 °C for long-term storage or transportation on dry ice to the clinical site. Prior to oral administration, the frozen enzyme solution was thawed and diluted to the necessary dosage strength. The placebo contained the identical buffer formulation as NHS7108 but excluded the enzyme component.

NHS7108 was evaluated in a 3-part, integrated phase 1a-1b clinical study, as outlined in Figure 6. Briefly, parts A and B investigated safety, tolerability, and PK following single ascending dose (SAD) and multiple ascending dose (MAD) in healthy adult subjects, respectively (Figure 6). Part C was a randomized, double-blind, placebo-controlled, single-dose, 2-way crossover part to investigate safety and PD of NHS7108 in subjects with severe EPI. Demographics and baseline characteristics were comparable between the study parts across the NHS7108 dose groups, and the

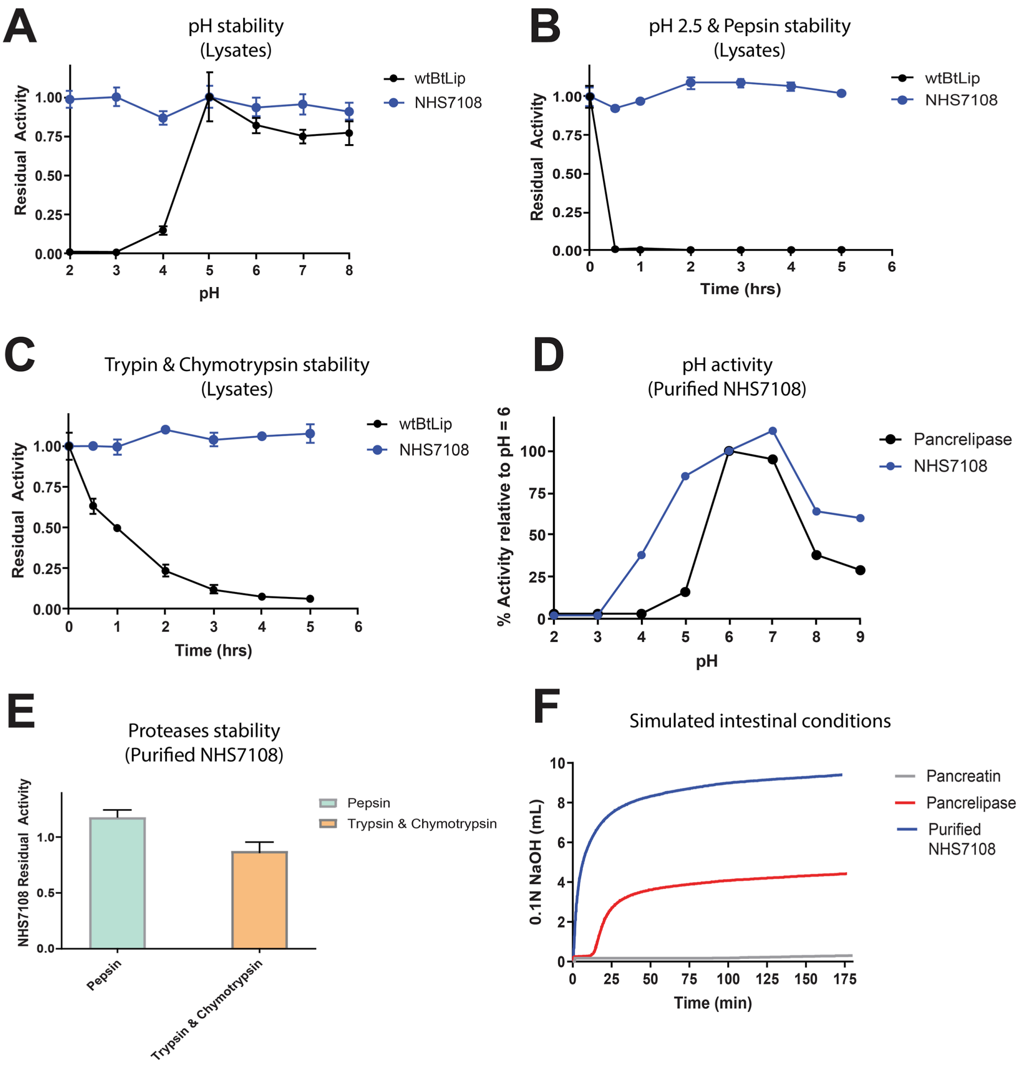

Figure 3. In vitro biochemical characterization of NHS7108. (A) Residual enzymatic activity after pH challenge for wtBtLip and NHS7108 lysates. The residual activity of each enzyme is normalized against its respective reference activity at pH 5.0. (B) Normalized residual enzymatic activity of each enzyme species upon incubation of the respective lysate with pepsin at pH 2.5. (C) Stability of wtBtLip and NHS7108 lysates against trypsin and chymotrypsin proteolysis. Catalytic activity is normalized to t = 0. (D) Percentage of catalytic activity of purified NHS7108 and pancrelipase as a function of pH (tested range 2.0–9.0) normalized to the measured activity at pH 6.0. (E) Stability of 1 mg/mL purified NHS7108 in the presence of either 1.5 mg/mL pepsin or trypsin/chymotrypsin, each at 1.5 mg/mL. Catalytic activity is normalized to t = 0. (F) Catalytic activity of purified NHS7108, enterically coated pancrelipase and pancreatin upon simulated ingestion. The graph shows the activity of each species in the presence of trypsin and chymotrypsin employing the pH-stat method as described in the Methods section.

pooled placebo as shown in Figure 7 and Table 1. NHS7108 showed a favorable safety profile, with all treatment-emergent adverse events (TEAEs) being mild or moderate (Table 2). No severe or serious AEs or significant changes in laboratory or vital signs were observed. Most reported TEAEs were unrelated to NHS7108, with headache and nausea being the most frequent NHS7108-related TEAEs. Of note, no NHS7108-related TEAE were reported in EPI patients (part C) and there was no treatment discontinuation due to AE.

Pharmacokinetic assessment of NHS7108 treatment indicated that serum concentrations of the enzyme were below the limit of quantification, suggesting no systemic absorption of NHS7108 after oral administration. The pharmacodynamic profile of NHS7108-treated EPI subjects (part C) was evaluated by the Pancreo-Lip breath test.[30] This surrogate assay offers an accurate and noninvasive approach for assessing the hydrolysis of triglycerides by an exogenous lipase in EPI patients within clinical settings.[29] The analysis demonstrated that lipid absorption in subjects from part C improved following a single dose of 150,000 units of NHS7108. More specifically, the mean 240_{min} cumulative $^{13}CO_2$ excretion in the NHS7108 group (14.8% dose) was 72% higher than that in the placebo group (8.6%

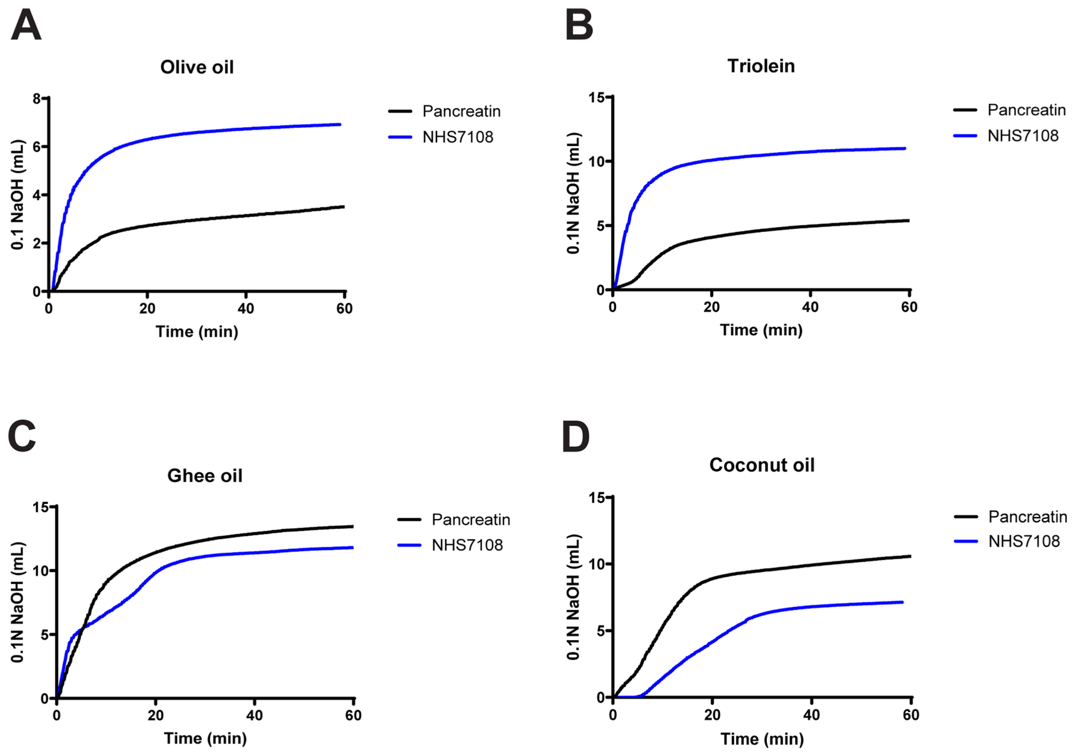

Figure 4. Activity of NHS7108 (blue trace) and pancreatin lipase (black trace) on different dietary fats as indicated in each panel; (A) olive oil, (B) triolein, (C) ghee oil, and (D) coconut oil.

dose) (Figure 8A). The mean percentage 150_{min} $^{13}CO_2$ excretion rate in the NHS7108 150,000 LU group (4.3% dose/hour) was 1.5 times higher than that in the placebo group (2.8% dose/hour) (Figure 8B). The curve of the percentage $^{13}CO_2$ excretion rates over baseline in the NHS7108 group showed a steeper increase than in the placebo group and continued to rise to a maximum of 5.5% dose/hour at 240 min postdose. The PancreoLip breath test values measured at screening (after PERT wash-out) were comparable to those measured in the placebo group. Collectively, the clinical findings show that NHS7108 lipase is safe, effectively digests fat in vivo, and enhances lipid absorption when taken orally in liquid form.

Discussion

EPI is a chronic condition characterized by decreased secretion or activity of pancreatic digestive enzymes which leads to clinical manifestations such as steatorrhea, weight loss, malabsorption, and maldigestion. Prevalence of EPI in the general population is largely unknown, but it is commonly estimated to be around 10%–20%, while it is perceived to be highly correlated with certain coconditions including CF, pancreatitis, cancer, and diabetes.[39] The standard of care for EPI is PERT, which, as of today, exclusively relies on the use of digestive enzymes extracted from porcine pancreas. However, PERT is associated with significant costs and compliance challenges, as EPI patients typically need to consume many pills daily to meet their digestive requirements.

Multiple attempts in the past focused on the study of recombinant lipases of microbial origins for the development of alternative PERTs.[14,16,18,19] Despite encouraging preclinical results, most of these efforts either did not undergo clinical testing or failed to achieve the %CFA end point in clinical trials.[40] The limited success observed thus far has been predominantly ascribed to the complicated nature of the digestive process. This complexity poses a significant challenge in finding a single microbial lipase capable of replacing the various lipases in pancreatic juice and effectively breaking down diverse triglycerides and fat-soluble vitamin esters. It is important to note that in previous studies, key phenotypic properties of recombinant lipases, such as low pH stability and resistance to proteolysis, were not optimized through engineering processes. Instead, only wild-type lipases were evaluated, with the sole exception being ANG003 from anagram, an engineered lipase that performed well in a recent phase-1 study.[21]

Driven by the medical need to develop an alternative recombinant-lipase-based PERT, a promiscuous microbial lipase was identified and its pH stability and resistance to proteolysis were optimized by engineering using Codexis' directed evolution platform CodeEvolver.[22,23] Using the triacylglycerol lipase from the thermophilic bacterium *B thermoamylovorans* (wtBtLip) as a starting template for engineering we isolated NHS7108 as the lead candidate for further characterization and development. The mutations

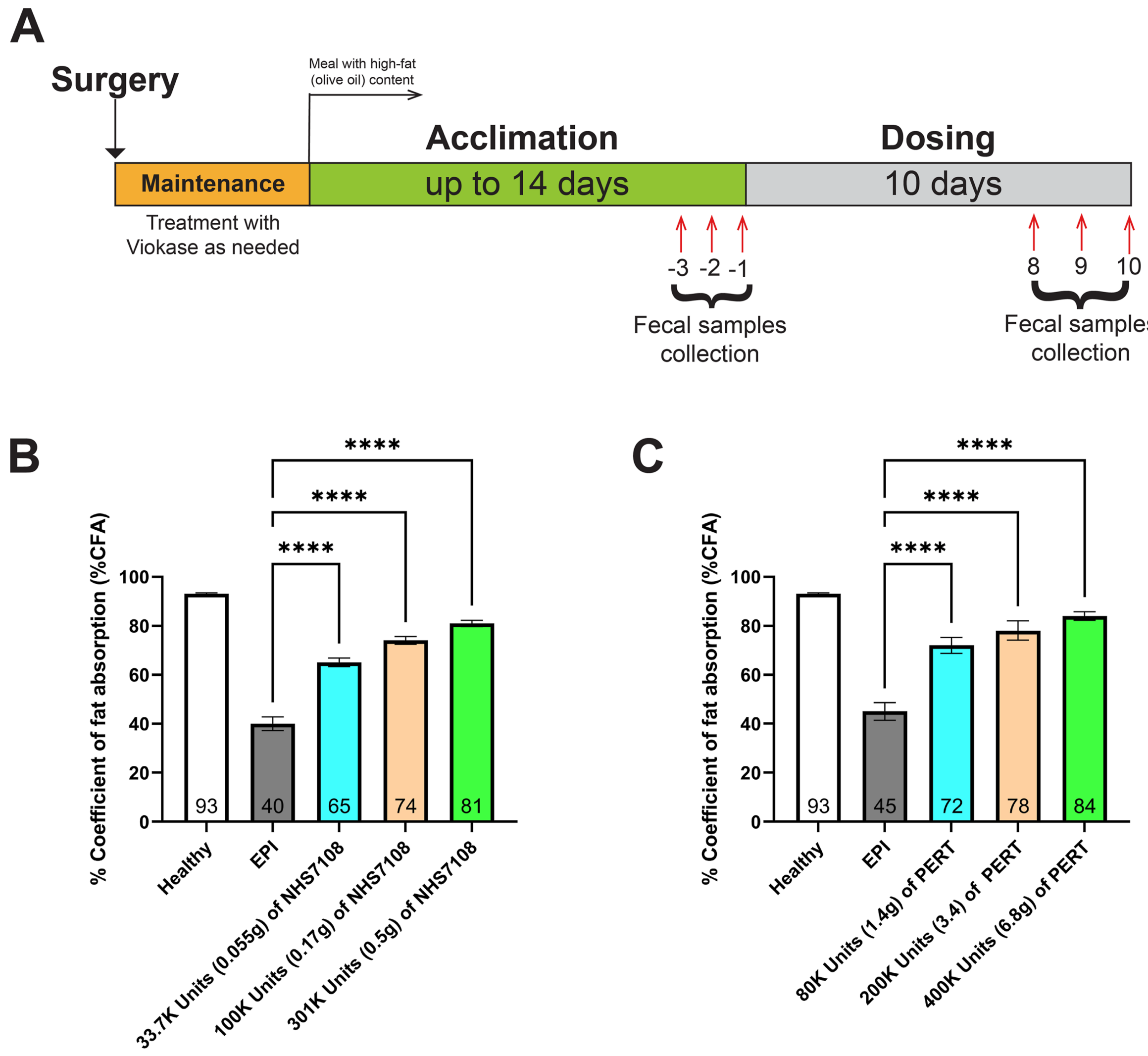

Figure 5. Dose-dependent effects of NHS7108 and PERT in a PDL minipig model. (A) The study design implemented to treat the PDL animals (detailed in the Methods section). (B) A graph showing the calculated %CFA vs each treatment with NHS7108 for the PDL minipigs. (C) %CFA vs PERT dose response in the PDL minipig animals. The values at the bottom of the bars indicate the %CFA value. Data are represented as mean ± SEM. Statistical significance was calculated by 1-way analysis of variance with Tukey posttest compared to the predose. ****$P < .0001$.

introduced during the engineering process, along with selection pressure by using simulated gastric and intestinal fluids as a surrogate end point assay, led to increased stability and activity at low pH levels and resistance to degradation by pepsin, trypsin, and chymotrypsin. Although it is extremely difficult to pinpoint the beneficial impact of each individual or combined mutations on the overall phenotypic improvement of NHS7108, it is reasonable to infer that the new residues collectively altered the physicochemical properties of the enzyme and its interaction and recognition by proteases (considering both primary sequence and structural conformations).

The enhanced stability of NHS7108, along with its broad hydrolyzing activity against various dietary fats, suggests that it is a promising candidate for a novel PERT. Importantly, NHS7108 demonstrates significantly broader pH activity compared to current PERT, exhibiting approximately 5 times greater activity at pH 5.0 (Figure 3D). This characteristic may result in enhanced enzyme efficacy under in vivo intestinal conditions for patients with EPI, who are reported to experience extended periods of postprandial intestinal pH $\leq$ 5 relative to healthy individuals.[41,42] NHS7108 would also be capable of effectively functioning in the GI tract without requiring enteric coating formulations which eliminates the need for expensive manufacturing processes and can also lead to slow kinetic enzyme release in the duodenum.[3] At postprandial pH 4.0, unlike PERT that was totally inactive, NHS7108 exhibited approximately 35% of its maximal activity (Figure 3D), indicating that, following meal ingestion, the enzyme may be active and contribute to lipid hydrolysis in the gastric compartment.

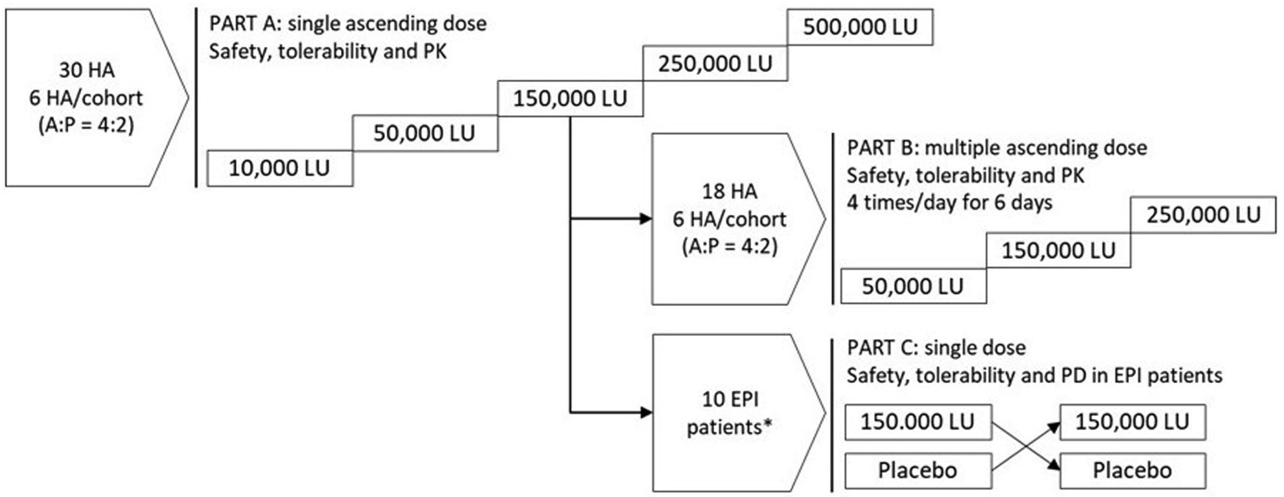

Figure 6. Clinical study design. HA, healthy adults; A, active; P, Placebo; PK, pharmacokinetics; EPI, exocrine pancreatic insufficiency; LU, lipase units. *Recruitment was stopped at 6 subjects instead of the planned 10 subjects, as the results of the interim analysis provided sufficient data to meet the planned objectives for Part C.

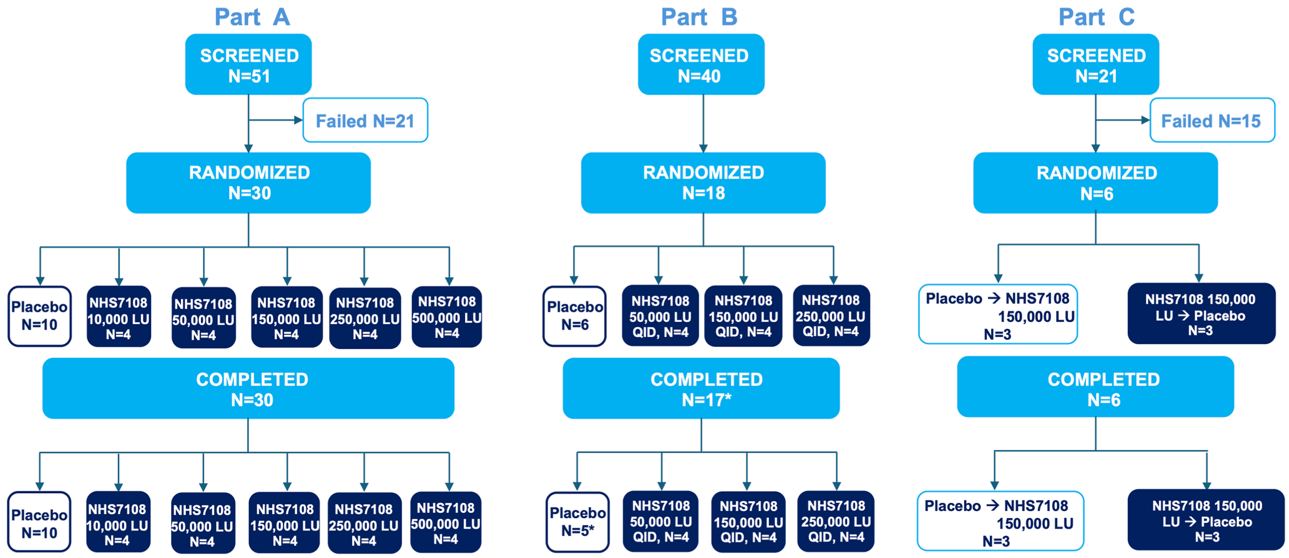

Figure 7. Patient disposition. *One participant receiving placebo in Part B discontinued due to COVID-19 (unrelated to study treatment). The recruitment in part C was stopped following the interim analysis on the first 5 subjects, as the results provided sufficient data to meet the planned objectives. Overall, 6 subjects were recruited instead of the 10 originally planned.

Table 1. Subject Demographics

Characteristic	Part A N = 30	Part B N = 18	Part C N = 6
Age, mean, y (range)	29 (19–55)	31 (19–48)	62 (43–73)
Male, n (%)	15 (50.0)	8 (44.4)	2 (33.3)
Female, n (%)	15 (50.0)	10 (55.6)	4 (66.7)
Ethnicity, n (%)			
White	22 (73.3)	14 (77.8)	5 (83.3)
Not Hispanic/Latino	29 (96.7)	14 (77.8)	6 (100.0)
BMI, mean kg/m^2 (SD)	24.0 (3.3)	25.2 (3.2)	22.9 (3.2)

BMI, body mass index; SD, standard deviation.

We subsequently assessed the performance of NHS7108 in both preclinical and clinical settings. Administration of the enzyme to PDL minipigs restored their ability to digest fat, as evidenced by the return of %CFA values to nearly healthy levels. Notably, NHS7108 returned %CFA to healthy levels by using ∼10-fold less amount by mass relative to pancrelipase-based PERT. This result demonstrates the potential of attaining high clinical efficacy with a significantly reduced quantity of active pharmaceutical ingredient, which may substantially decrease pill burden and consequently enhance patient compliance. Most importantly, in an integrated phase 1a-1b clinical trial

Table 2. Safety Results by Study Part, Dose Group, and Treatment Allocation

	Part A N = 30							Part B N = 18					Part C N = 6		
	Pooled PBO (N = 10)	NHS7108 dose groups (LU)					All adults (N = 30)	Pooled PBO (N = 6)	NHS7108 dose groups (LU)			All adults (N = 18)	PBO (N = 6)	NHS7108 150,000 LU (N = 6)	All adults (N = 6)
		10,000	50,000	150,000	250,000	500,000			50,000	150,000	250,000				
≥1 TEAE, n(%), E	2 (20.0), 3	1 (25.0), 1	3 (75.0), 4	2 (50.0), 3	1 (25.0), 1	3 (75.0), 3	12 (40.0), 15	5 (83.3), 12	4 (100.0), 13	3 (75.0), 7	3 (75.0), 5	15 (83.3), 37	5 (83.3), 6	1 (16.7), 2	5 (83.3), 8
≥1 intervention-related TEAE, n(%), E	1 (10.0), 2	0	2 (50.0), 3	2 (50.0), 3	0	0	5 (16.7)	1 (16.7), 1	3 (75.0), 4	1 (25.0), 2	0	5 (27.8), 7	1 (16.7), 1	0	1 (16.7), 1
TEAE by maximum severity															
Mild	2 (20.0)	1 (25.0)	3 (75.0)	2 (50.0)	1 (25.0)	2 (50.0)	11 (36.7)	4 (66.7)	3 (75.0)	3 (75.0)	3 (75.0)	13 (72.2)	5 (83.3)	1 (16.7)	5 (83.3)
Moderate	0	0	0	0	0	1 (25.0)	1 (3.3)	1 (16.7)	1 (25.0)	0	0	2 (11.1)	0	0	0
Severe	0	0	0	0	0	0	0	0	0	0	0	0	0	0	0
Intervention-related TEAE by maximum severity															
Mild	1 (10.0)	0	2 (50.0)	2 (50.0)	0	0	5 (16.7)	1 (16.7)	3 (75.0)	1 (25.0)	0	5 (27.8)	1 (16.7)	0	1 (16.7)
Moderate	0	0	0	0	0	0	0	0	0	0	0	0	0	0	0
Severe	0	0	0	0	0	0	0	0	0	0	0	0	0	0	0

E, number of subjects; LU, lipase units; PBO, placebo; TEAE, treatment-emergent adverse event.

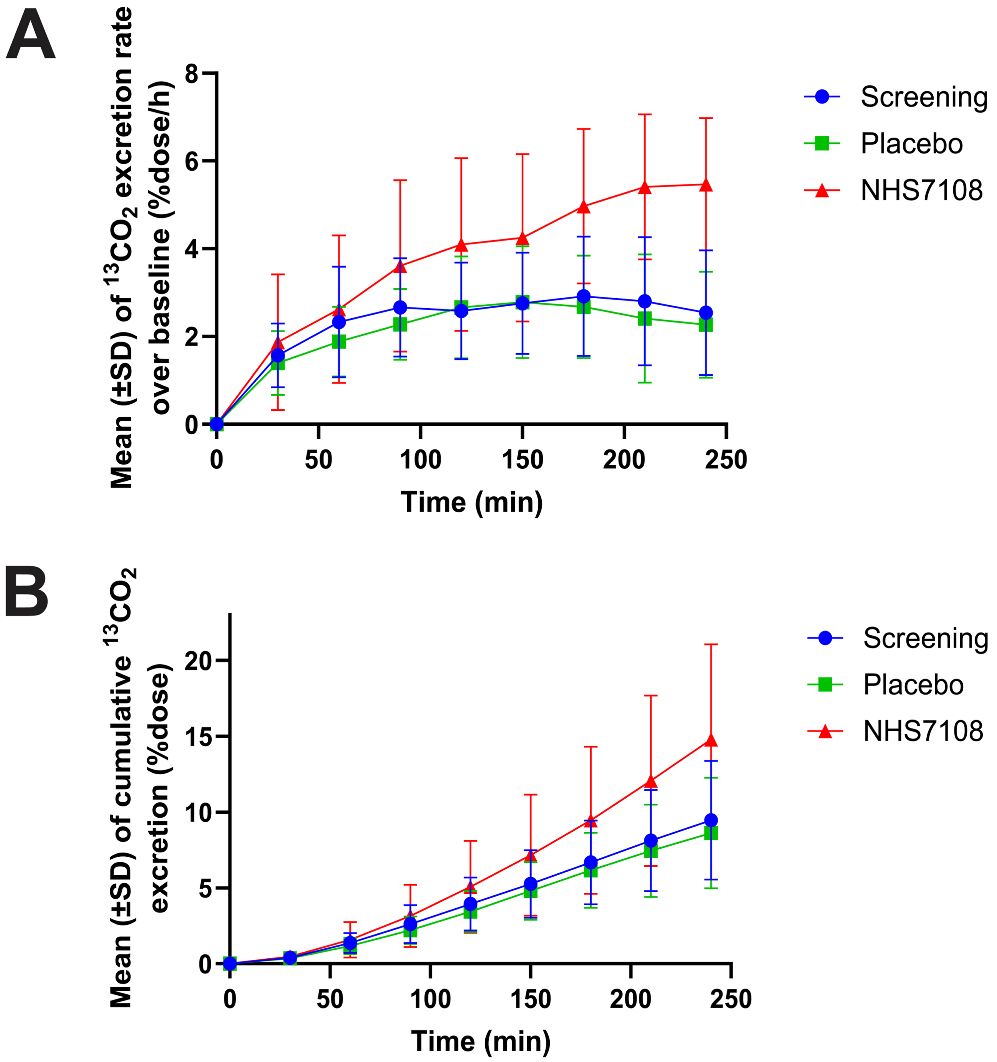

Figure 8. Pharmacodynamic evaluation of 6 patients with EPI following a single oral administration of 150,000 units of NHS7108. (A) Percentage (%dose/h) of $^{13}CO_2$ excretion rate over baseline. (B) Area under the curve mean cumulative $^{13}CO_2$ excretion (%dose) indicative of the lipid absorption, as measured by the breath test. In both graphs the screening, placebo, and NHS7108 treatments are shown in blue, green, and red traces, respectively. Data are shown as means ± SD values. SD, standard deviation.

assessing the safety, PK, and PD of oral NHS7108, the enzyme did not cause any severe AEs, which were similar to those observed with the placebo, and it was not detected in the serum of the dosed subjects. Of particular importance, the enzyme demonstrated a promising pharmacodynamic profile, as shown by a proof-of-concept breath test end point that indirectly correlates well with how effectively fat is absorbed.[30,43] However, directly comparing our data to standard-of-care PERT is challenging because different trials have used different efficacy end points—approved PERTs typically use %CFA, whereas our exploratory study used a breath test. One limitation of our study pertains to the relatively small cohort of EPI patients (n = 6) included in the proof-of-concept phase 1a-1b trial. Given the exploratory design, the sample size was not determined by formal statistical considerations, and all statistical analyses were descriptive in nature and not intended to demonstrate statistical power. Collectively, these findings indicate that NHS7108 may potentially be an additional component for EPI treatment and merits further clinical testing in a larger trial with a solid formulation, in conjunction with ongoing development of complementary protease/amylase combination approaches.

Conclusion

At present, the standard treatment for EPI is PERT, which relies exclusively on pancrelipase sourced from porcine pancreatic extracts. Despite its benefits, pancrelipase presents certain limitations and potential risks, underscoring the clinical significance for the development of high-potency recombinant digestive enzymes. Our early findings indicate that the engineered NHS7108 microbial lipase may provide an effective nonporcine treatment option for EPI patients, as evidenced by improved fat absorption and a favorable safety profile in a proof-of-concept phase 1a-1b trial.

One limitation of our study is related to the relatively small cohort of EPI patients (n = 6) included in the phase 1a-1b trial. Due to the exploratory nature of the study, sample size was not established based on formal statistical criteria. All statistical analyses performed were descriptive

and not intended to demonstrate statistical power. Collectively, these results suggest that NHS7108 could be a useful addition to EPI treatment and warrants further clinical trials with a solid formulation, alongside efforts to develop protease/amylase combinations.

Supplementary Materials

Material associated with this article can be found, in the online version, at https://doi.org/10.1016/j.gastha.2026.100984.

References

1. Capurso G, Traini M, Piciucchi M, et al. Exocrine pancreatic insufficiency: prevalence, diagnosis, and management. Clin Exp Gastroenterol 2019;12:129–139.
2. Trang T, Chan J, Graham DY. Pancreatic enzyme replacement therapy for pancreatic exocrine insufficiency in the 21st century. World J Gastroenterol 2014; 20:11467.
3. Fieker A, Philpott J, Armand M. Enzyme replacement therapy for pancreatic insufficiency: present and future. Clin Exp Gastroenterol 2011;4:55–73.
4. Min M, Patel B, Han S, et al. Exocrine pancreatic insufficiency and malnutrition in chronic pancreatitis: identification, treatment, and consequences. Pancreas 2018;47:1015.
5. Ketwaroo GA, Graham DY. Rational use of pancreatic enzymes for pancreatic insufficiency and pancreatic pain. Adv Exp Med Biol 2019;1148:323–343.
6. Stead RJ, Skypala I, Hodson ME. Treatment of steatorrhea in cystic fibrosis: a comparison of enteric-coated microspheres of pancreatin versus non-enteric-coated pancreatin and adjuvant cimetidine. Aliment Pharmacol Ther 1988;2:471–482.
7. Layer P, Keller J, Lankisch PG. Pancreatic enzyme replacement therapy. Curr Gastroenterol Rep 2001; 3:101–108.
8. Meyer JH, Gu YG, Jehn D, et al. Factors that affect the performance of lipase on fat digestion and absorption in a canine model of pancreatic insufficiency. Pancreas 1994;9:613–623.
9. Aloulou A, Puccinelli D, Sarles J, et al. In vitro comparative study of three pancreatic enzyme preparations: dissolution profiles, active enzyme release and acid stability. Aliment Pharmacol Ther 2008;27:283–292.
10. DiMagno EP, Malagelada JR, Go VLW, et al. Fate of orally ingested enzymes in pancreatic insufficiency. Comparison of two dosage schedules. N Engl J Med 1977;296:1318–1322.
11. Domínguez-Muñoz JE, Iglesias-García J, Iglesias-Rey M, et al. Effect of the administration schedule on the therapeutic efficacy of oral pancreatic enzyme supplements in patients with exocrine pancreatic insufficiency: a randomized, three-way crossover study. Aliment Pharmacol Ther 2005;21:993–1000.
12. Shandro BM, Nagarajah R, Poullis A. Challenges in the management of pancreatic exocrine insufficiency. World J Gastrointest Pharmacol Ther 2018;9:39–46.
13. Barkin JA, Delk TB, Powell VJ. Symptoms, burden, and unmet needs of patients living with exocrine pancreatic insufficiency: a narrative review of the patient experience. BMC Gastroenterol 2024;24:101.
14. Lowe ME, Whitcomb DC. Next generation of pancreatic enzyme replacement therapy: recombinant microbial enzymes and finding the perfect lipase. Gastroenterology 2015;149:1678–1681.
15. Freedman SD, Zaworski K, Pierzynowska K, et al. Validation of an omega-3 substrate challenge absorption test as an indicator of global fat lipolysis. PLoS One 2023;18:e0284651.
16. Anon. Yarrowia lipolytica lipase 2 is stable and highly active in test meals and increases fat absorption in an animal model of pancreatic exocrine insufficiency - PubMed. Available at: https://pubmed-ncbi-nlm-nih-gov.ezproxy.lib.utexas.edu/26327131/. Accessed November 8, 2024.
17. Kurfürst M, Friedel R, Friedrich O, et al. Pharmaceutical preparation comprising lipase of bacterial origin, 2010. Available at: https://patents.google.com/patent/WO2010085975A1/en. Accessed November 8, 2024.
18. Brock A, Aldag I, Edskes S, et al. Novel ciliate lipases for enzyme replacement during exocrine pancreatic insufficiency. Eur J Gastroenterol Hepatol 2016;28:1305–1312.
19. Borowitz D, Stevens C, Brettman LR, et al. Liprotamase long-term safety and support of nutritional status in pancreatic-insufficient cystic fibrosis. J Pediatr Gastroenterol Nutr 2012;54:248–257.
20. Borowitz D, Stevens C, Brettman LR, et al. International phase III trial of liprotamase efficacy and safety in pancreatic-insufficient cystic fibrosis patients. J Cyst Fibros 2011;10:443–452.
21. Sathe M, Freedman SD, Putman MS, et al. Results of a clinical trial of ANG003, a non-porcine pancreatic enzyme replacement therapy, in people with cystic fibrosis. J Cyst Fibros 2025;24:1043–1050.
22. Chng C, Hallows WC, Viduya JVA, et al. Engineered lipase variants, 2021. Available at: https://patents.google.com/patent/WO2021041809A1/en?oq=WO2021%2f041809+A1. Accessed December 11, 2024.
23. Skvorak K, Mitchell V, Teadt L, et al. An orally administered enzyme therapeutic for homocystinuria that suppresses homocysteine by metabolizing methionine in the gastrointestinal tract. Mol Genet Metab 2023;139:107653.
24. Karamitros CS, Murray K, Kumada Y, et al. Mechanistic conformational and substrate selectivity profiles emerging in the evolution of enzymes via parallel trajectories. Nat Commun 2024;15:7068.
25. Karamitros CS, Murray K, Winemiller B, et al. Leveraging intrinsic flexibility to engineer enhanced enzyme catalytic activity. Proc Natl Acad Sci U S A 2022;119: e2118979119.
26. Hooft RW, Vriend G, Sander C, et al. Errors in protein structures. Nature 1996;381:272.
27. Gregory PC, Hoffmann K, Kamphues J, et al. The pancreatic duct ligated (Mini)pig as a model for pancreatic exocrine insufficiency in man. Pancreas 2016;45:1213–1226.
28. Kelleher J, Walker BE, Losowsky MS. An assessment of the Van de Kamer method for estimation of faecal fat. Clin Chim Acta 1970;30:267–271.

29. Löser C, Brauer C, Aygen S, et al. Comparative clinical evaluation of the 13C-mixed triglyceride breath test as an indirect pancreatic function test. Scand J Gastroenterol 1998;33:327–334.
30. Anon. 13C-mixed triglyceride breath test to assess oral enzyme substitution therapy in patients with chronic pancreatitis - PubMed. Available at: https://pubmed.ncbi.nlm.nih.gov/17445754/. Accessed December 11, 2024.
31. Matsumura H, Yamamoto T, Leow TC, et al. Novel cation-pi interaction revealed by crystal structure of thermoalkalophilic lipase. Proteins 2008;70:592–598.
32. Jeong S-T, Kim H-K, Kim S-J, et al. Novel zinc-binding center and a temperature switch in the Bacillus stearothermophilus L1 lipase. J Biol Chem 2002; 277:17041–17047.
33. Gihaz S, Kanteev M, Pazy Y, et al. Filling the void: introducing aromatic interactions into solvent tunnels to enhance lipase stability in methanol. Appl Environ Microbiol 2018;84:e02143-18.
34. Jaeger KE, Dijkstra BW, Reetz MT. Bacterial biocatalysts: molecular biology, three-dimensional structures, and biotechnological applications of lipases. Annu Rev Microbiol 1999;53:315–351.
35. Yamada C, Sawano K, Iwase N, et al. Isolation and characterization of a thermostable lipase from Bacillus thermoamylovorans NB501. J Gen Appl Microbiol 2017; 62:313–319.
36. Fazio TN, Healy L, Heise T, et al. Pharmacodynamics, safety, tolerability and pharmacokinetics of a single oral dose of an engineered phenylalanine ammonia-lyase in patients with phenylketonuria. Mol Genet Metab Rep 2023;37:101012.
37. Majtan T, Kožich V, Kruger WD. Emerging therapeutic approaches to cystathionine beta-synthase-deficient homocystinuria. Br J Pharmacol 2022;180:264.
38. Krieger E, Joo K, Lee J, et al. Improving physical realism, stereochemistry and side-chain accuracy in homology modeling: four approaches that performed well in CASP8. Proteins 2009;77:114.
39. Lewis D. An updated review of exocrine pancreatic insufficiency prevalence finds EPI to be more common in general population than rates of co-conditions. J Gastrointestin Liver Dis 2024;33:123–130.
40. Konstan MW, Wagener JS, Wilschanski M, et al. A comparison of liprotamase, a non-porcine pancreatic enzyme replacement therapy, to porcine extracted pancrelipase in a noninferiority randomized clinical trial in patients with cystic fibrosis. Clin Investig 2018;8:147–154.
41. Geus WP, Eddes EH, Gielkens HA, et al. Post-prandial intragastric and duodenal acidity are increased in patients with chronic pancreatitis. Aliment Pharmacol Ther 1999;13:937–943.
42. Youngberg CA, Berardi RR, Howatt WF, et al. Comparison of gastrointestinal pH in cystic fibrosis and healthy subjects. Dig Dis Sci 1987;32:472–480.
43. Domínguez-Muñoz JE, Nieto L, Vilariño M, et al. Development and diagnostic accuracy of a breath test for pancreatic exocrine insufficiency in chronic pancreatitis. Pancreas 2016;45:241–247.

Received October 31, 2025. Accepted April 17, 2026.

Correspondence:
Address correspondence to: Christos S. Karamitros, MSc, PhD, Nestlé Health Science, Rte du Jorat 57, Lausanne 1000, Switzerland. e-mail: christos.karamitros@us.nestle.com.

Acknowledgments:
The authors thank Charlene Ching, Melissa Mayo, David Stanley, Jackie Fleming, Ann Lao, Anna Zibert, Vivian Chang, Nianliu Zhang, Long Pang, Daley Nguyen, Monica Ramel, Riley Curd, Thuan La, Stephanie Blum, Maximilian von Eynatten, and Hans-Juergen Woerle.

Authors' Contributions:
Christos S. Karamitros: Design of animal studies, protein engineering and biochemical data interpretation, led the drafting & preparation of the manuscript. Chinping Chng: Execution of biochemical characterization. William Casey Hallows: Characterization of initial lipases pool, biochemical assay development, screening of lipase variants. Ravi Garcia: Screening of the lipase variants, assay development and performed biochemical characterization. Kristen Skvorak: Design and management of animal studies. Adam Silverman: Oversaw biochemical characterization of 7108. Nikki Kruse: Gene acquisition that identified the wild-type lipase, bioinformatics support, library designs for enzyme engineering. Judy Viduya: Assay development, screening of lipase variants. Stephanie Galanie: Developed the RapidFire Mass Spec. assay that enabled all the HTP screenings of lipase variants. Da Duan: Performed pH stat experiments to guide dosing for in-vivo studies. Kerryn McCluskie: Guidance on in-vivo study design and data analysis. Robert K. Sato: CMC Lead. John Watson: Design & management of animal studies. Alek Zajac: Served as the Project Lead of the project. Carmine D'Urzo: Clinical Development Lead and design of the Phase 1 study. Sharan Ghuman: Clinical operations & execution of Phase 1 study. Edward Edson: Clinical operations & execution of Phase 1 study. Ismail Aouadi: Biostatistical analysis. Chris Wynne: Phase 1 study investigator. John Windsor: Phase 1 study investigator. Gjalt Huisman: Executive supervision of the program. Bernard Cuenoud: Oversaw early preclinical development activities.

Conflicts of Interest:
The authors disclose no conflicts.

Funding:
The authors report no funding.

Ethical Statement:
The nonclinical and clinical studies were approved by the respective ethics committees as imposed by regulatory guidelines.

Data Transparency Statement:
Raw data can be shared upon reasonable request.

Reporting Guidelines:
SAGER.

Gastro Hep Advances, Volume 5

Supplemental information

Development of a Potent Engineered Microbial Lipase for the Treatment of Exocrine Pancreatic Insufficiency

Christos S. Karamitros, Chinping Chng, William Casey Hallows, Ravi Garcia, Kristen Skvorak, Adam Silverman, Nikki Kruse, Judy Viduya, Stephanie Galanie, Da Duan, Kerryn McCluskie, Robert K. Sato, John Watson, Alek Zajac, Carmine D'Urzo, Sharan Ghuman, Edward Edson, Ismail Aouadi, Chris Wynne, John Windsor, Gjalt Huisman, and Bernard Cuenoud

Supplementary Information

Development of a potent, engineered microbial lipase for the treatment of exocrine pancreatic insufficiency

Christos S. Karamitros[1*], Chinping Chng[2], William Casey Hallows[2], Ravi Garcia[2], Kristen Skvorak[2], Adam Silverman[2], Nikki Kruse[2], Judy Viduya[2], Stephanie Galanie[2], Da Duan[2], Kerryn McCluskie[2], Bob Sato[2],John Watson[1], Alek Zajac[1], Carmine D'Urzo[1], Sharan Ghuman[1], Edward Edson[1], Ismail Aouadi[1], Chris Wynne[3], John Windsor[4], Gjalt Huisman[2], Bernard Cuenoud[1]

[1]Nestle Health Science, Lausanne, 1000, Switzerland

[2]Codexis Inc., Redwood City, California, USA

[3]New Zealand Clinical Research, Christchurch, New Zealand

[4]University of Auckland, Auckland, New Zealand

***Corresponding author**

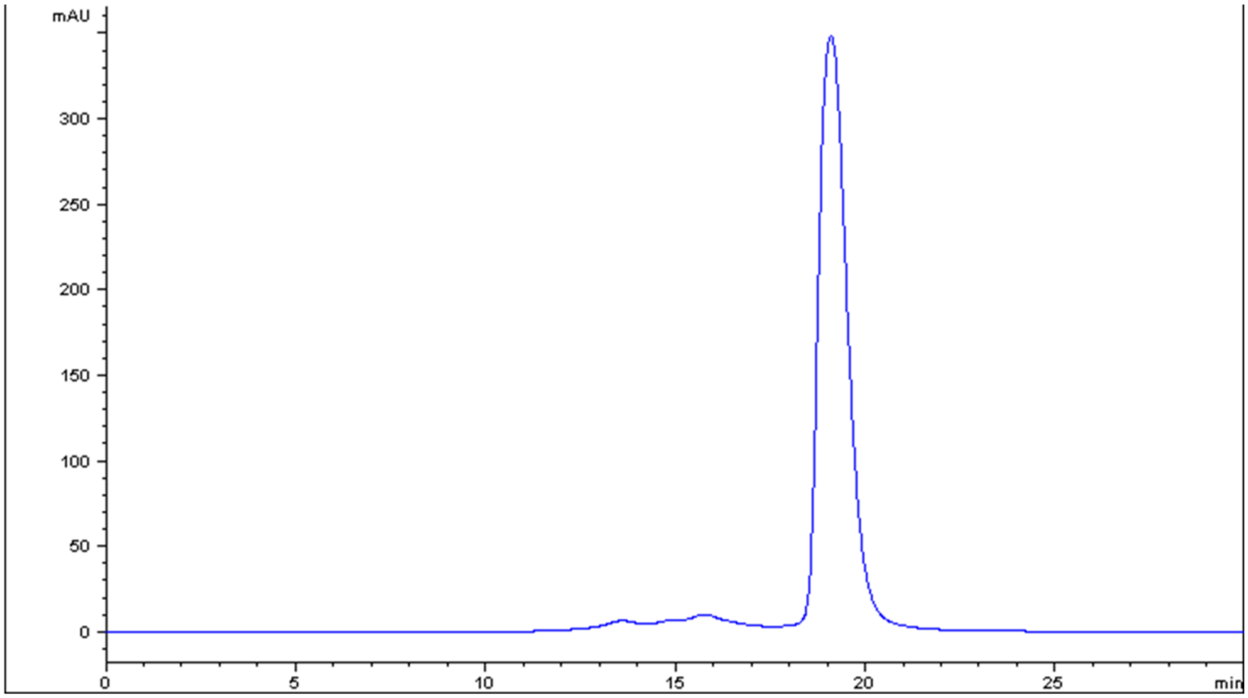

Peak	%Area A^{215}
HMW	9
NHS7108	91

Supplementary Figure 1. Analytical size-exclusion chromatogram (aSEC) of purified NHS7108. In the graph, Y- and X-axis represent the Absorbance^{215} and the retention time (minutes) respectively. HMW: High-Molecular-Weight species.

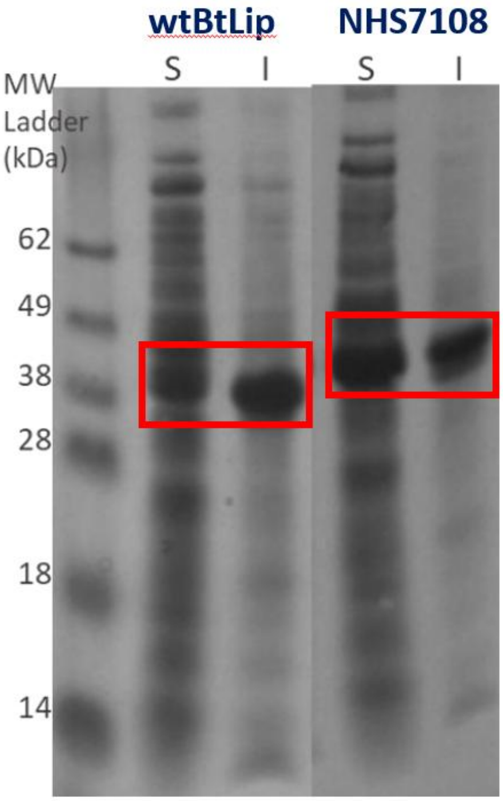

Supplementary Figure 2. SDS-PAGE of reconstituted enzyme powder of wtBtLip and NHS7108 expressed in *E. coli*. S and I indicate soluble and insoluble fractions whereas the red squares show the respective lipase bands (theoretical molecular weight: 43.6 kDa).

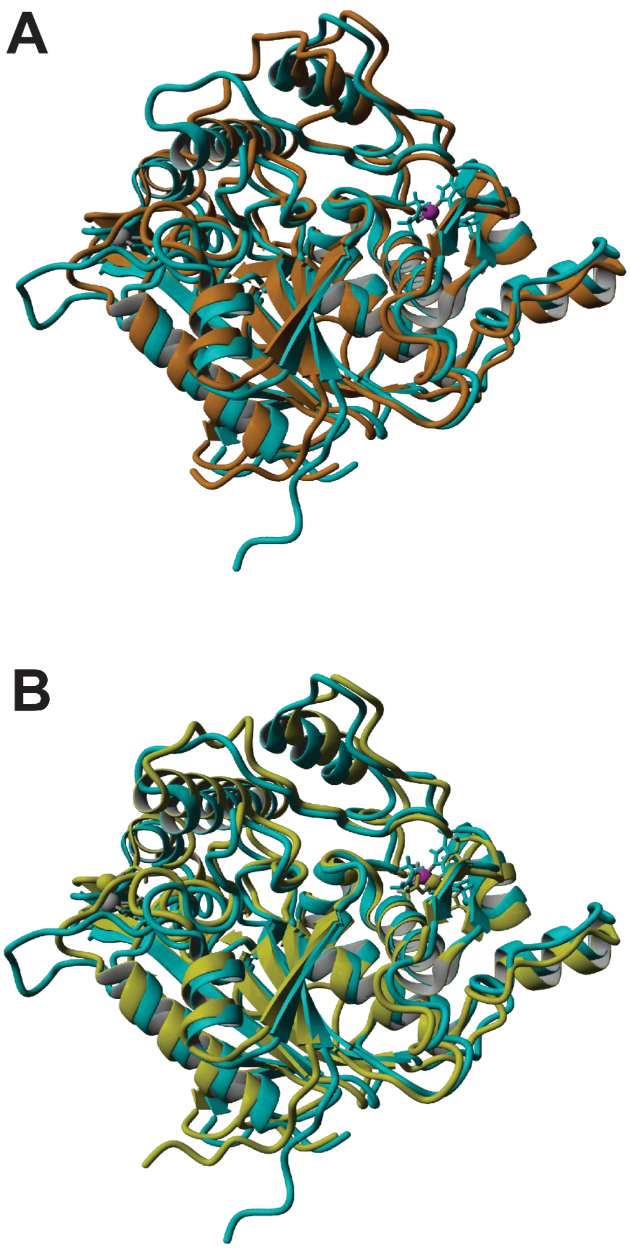

Supplementary Figure 3. NHS7108 homology model overlaid against structures of other microbial lipases which were experimentally determined by x-ray crystallography. (A) NHS7108 (cyan) vs T1 lipase (orange) from *G. zalihae* (PDB entry: 2DSN). (B) NHS7108 (cyan) against L1 lipase (yellow) from *B. stearothermophilus* (PDB entry: 1KU0) respectively. NHS7108 shares ~ 47% amino acid identity with each of the lipases whereas the RMSD of both structural alignments is ~5.3 Å. All the structural analysis in this study was done with the program YASARA Structure unless otherwise stated.

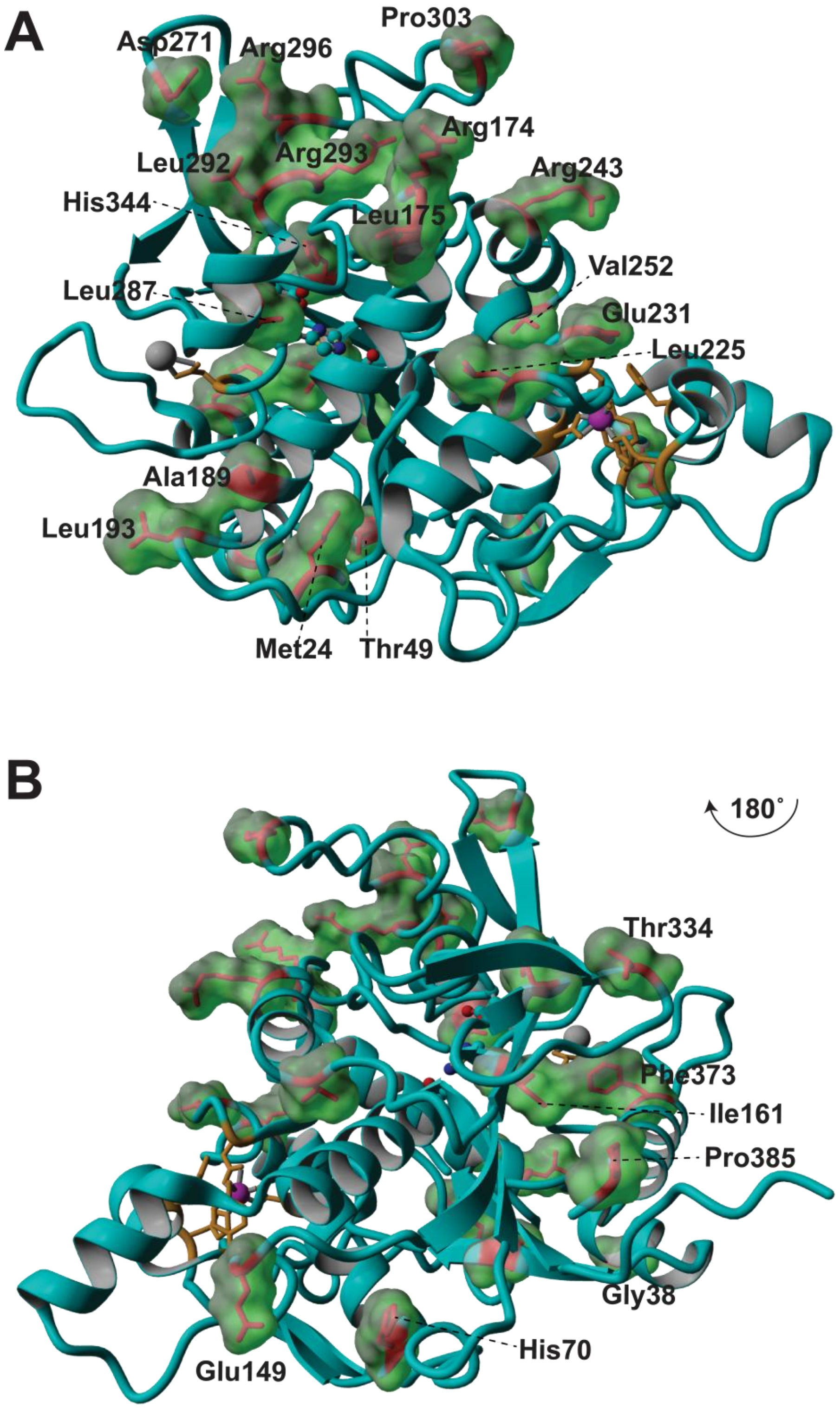

Supplementary Figure 4. (A) The modeled structure of NHS7108 displaying 24 amino acid substitutions, shown as red sticks and labeled in black. Residues interacting with Zn^{2+} (magenta sphere) and Ca^{2+} (grey sphere) metals are depicted in orange. (B) A 180º rotation of the structure is provided to enhance visualization of the amino acid substitutions at positions Gly38, His70, Glu149, Ile161, Thr334, Phe373, and Pro385.

Supplementary Table 1. Summary of the administration of various doses of NHS7108 and commercial PERT to PDL minipigs, along with the corresponding calculated %CFA values for each treatment group. Pre- and post-dose %CFA values were determined using data from the final count of animals per group as shown below, excluding minipigs with pre-dose %CFA above 60%. Data are represented as mean ± SEM. Δ is defined as ($\%CFA^{post\text{-}dose}$-$\%CFA^{pre\text{-}dose}$). (As outlined in the Methods section, purified NHS7108 was administered as lyophilized fermentation powder, while PERT was given in the form of enterically coated minitablets.

				%CFA			
**Treatment**	**Units/day (in 1000s)**	**Amount (g)**	**Final number of animals per group**	**Pre-surgery**	**Pre-dose**	**Post-dose**	**Δ**
NHS7108	33.7	0.055	2	93±1.5	40±4	65.5±2	25.5±6
	100	0.17	3		37.5±4	74±1	36.5±5
	301	0.5	3		42±6	81±1	39±7
PERT	80	1.4	4		50±7	72±3	22±10
	200	3.4	4		44±4	78±4	34±8
	400	6.8	4		44.5±4	84±2	39.5±6

Supplementary Information

Development of a potent, engineered microbial lipase for the treatment of exocrine pancreatic insufficiency

Christos S. Karamitros[1*], Chinping Chng[2], William Casey Hallows[2], Ravi Garcia[2], Kristen Skvorak[2], Adam Silverman[2], Nikki Kruse[2], Judy Viduya[2], Stephanie Galanie[2], Da Duan[2], Kerryn McCluskie[2], Bob Sato[2],John Watson[1], Alek Zajac[1], Carmine D'Urzo[1], Sharan Ghuman[1], Edward Edson[1], Ismail Aouadi[1], Chris Wynne[3], John Windsor[4], Gjalt Huisman[2], Bernard Cuenoud[1]

[1]Nestle Health Science, Lausanne, 1000, Switzerland

[2]Codexis Inc., Redwood City, California, USA

[3]New Zealand Clinical Research, Christchurch, New Zealand

[4]University of Auckland, Auckland, New Zealand

***Corresponding author**

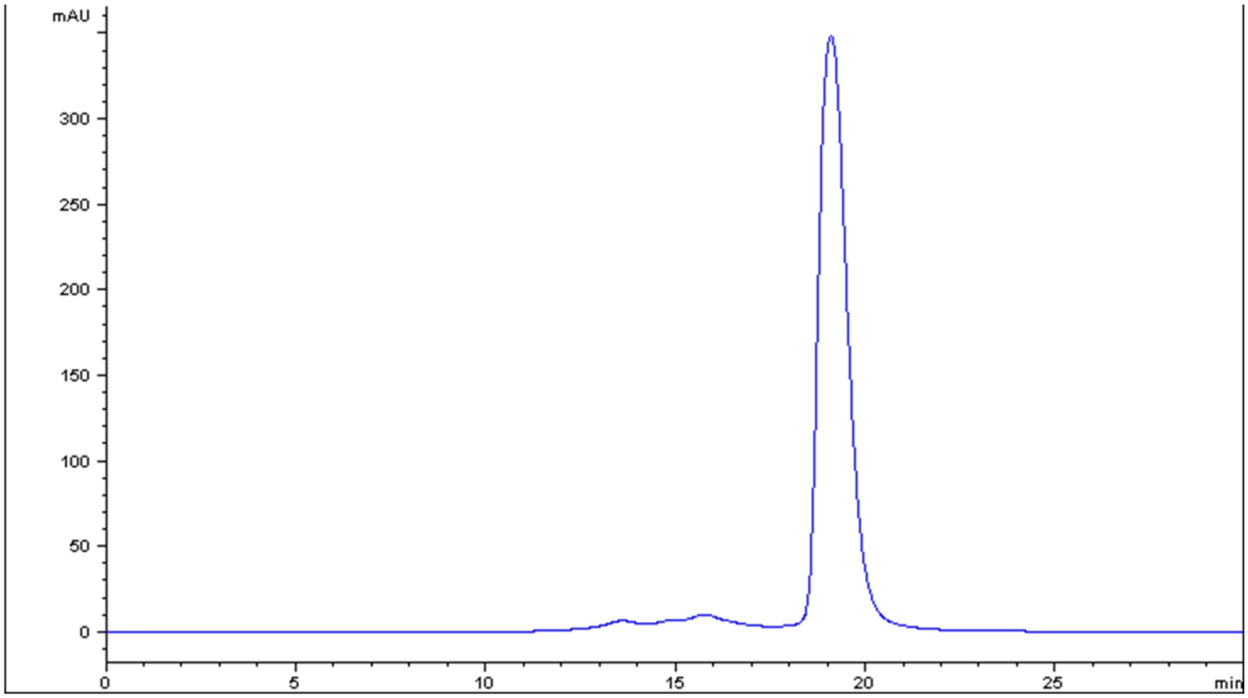

Peak	%Area A^{215}
HMW	9
NHS7108	91

Supplementary Figure 1. Analytical size-exclusion chromatogram (aSEC) of purified NHS7108. In the graph, Y- and X-axis represent the $Absorbance^{215}$ and the retention time (minutes) respectively. HMW: High-Molecular-Weight species.

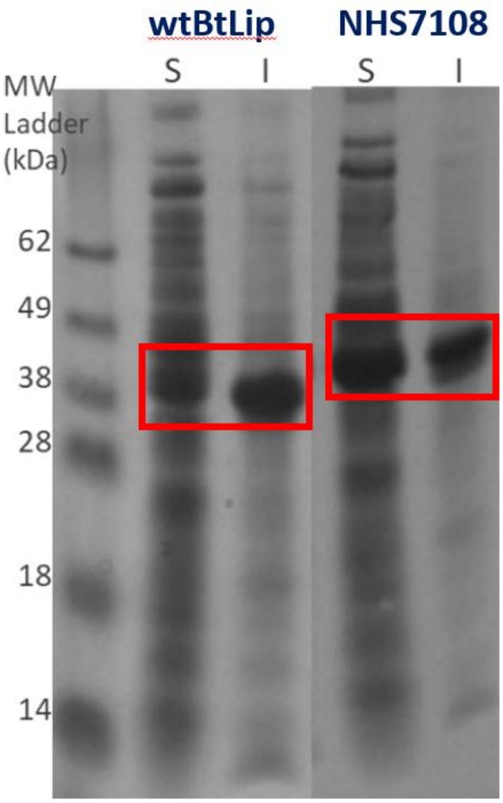

Supplementary Figure 2. SDS-PAGE of reconstituted enzyme powder of wtBtLip and NHS7108 expressed in *E. coli*. S and I indicate soluble and insoluble fractions whereas the red squares show the respective lipase bands (theoretical molecular weight: 43.6 kDa).

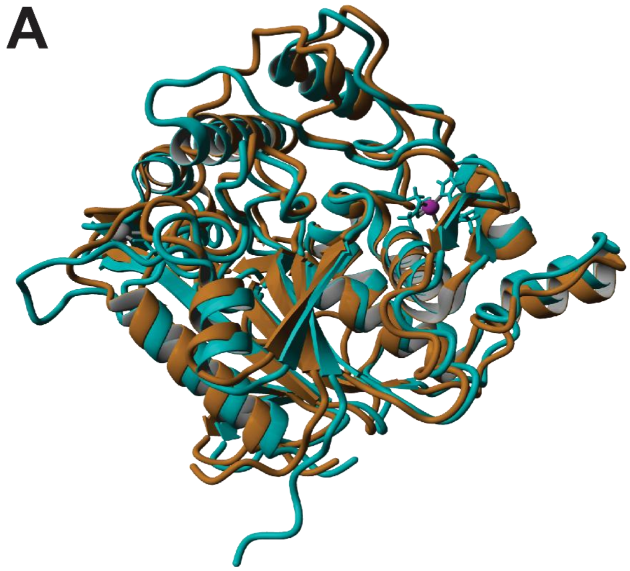

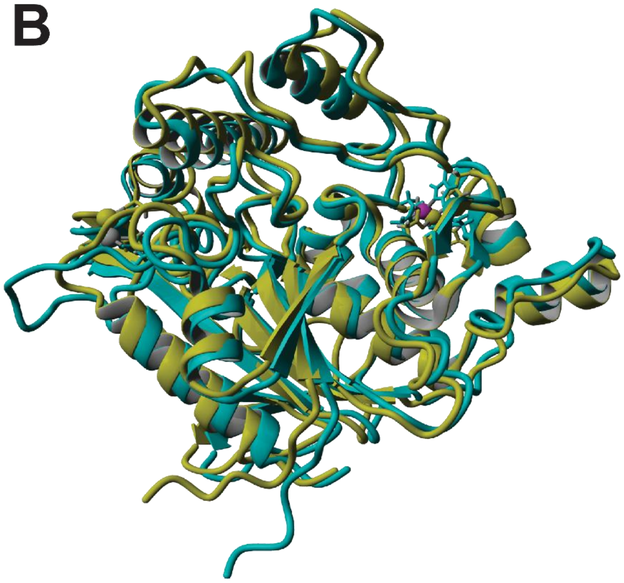

Supplementary Figure 3. NHS7108 homology model overlaid against structures of other microbial lipases which were experimentally determined by x-ray crystallography. (A) NHS7108 (cyan) vs T1 lipase (orange) from *G. zalihae* (PDB entry: 2DSN). (B) NHS7108 (cyan) against L1 lipase (yellow) from *B. stearothermophilus* (PDB entry: 1KU0) respectively. NHS7108 shares ~ 47% amino acid identity with each of the lipases whereas the RMSD of both structural alignments is ~5.3 Å. All the structural analysis in this study was done with the program YASARA Structure unless otherwise stated.

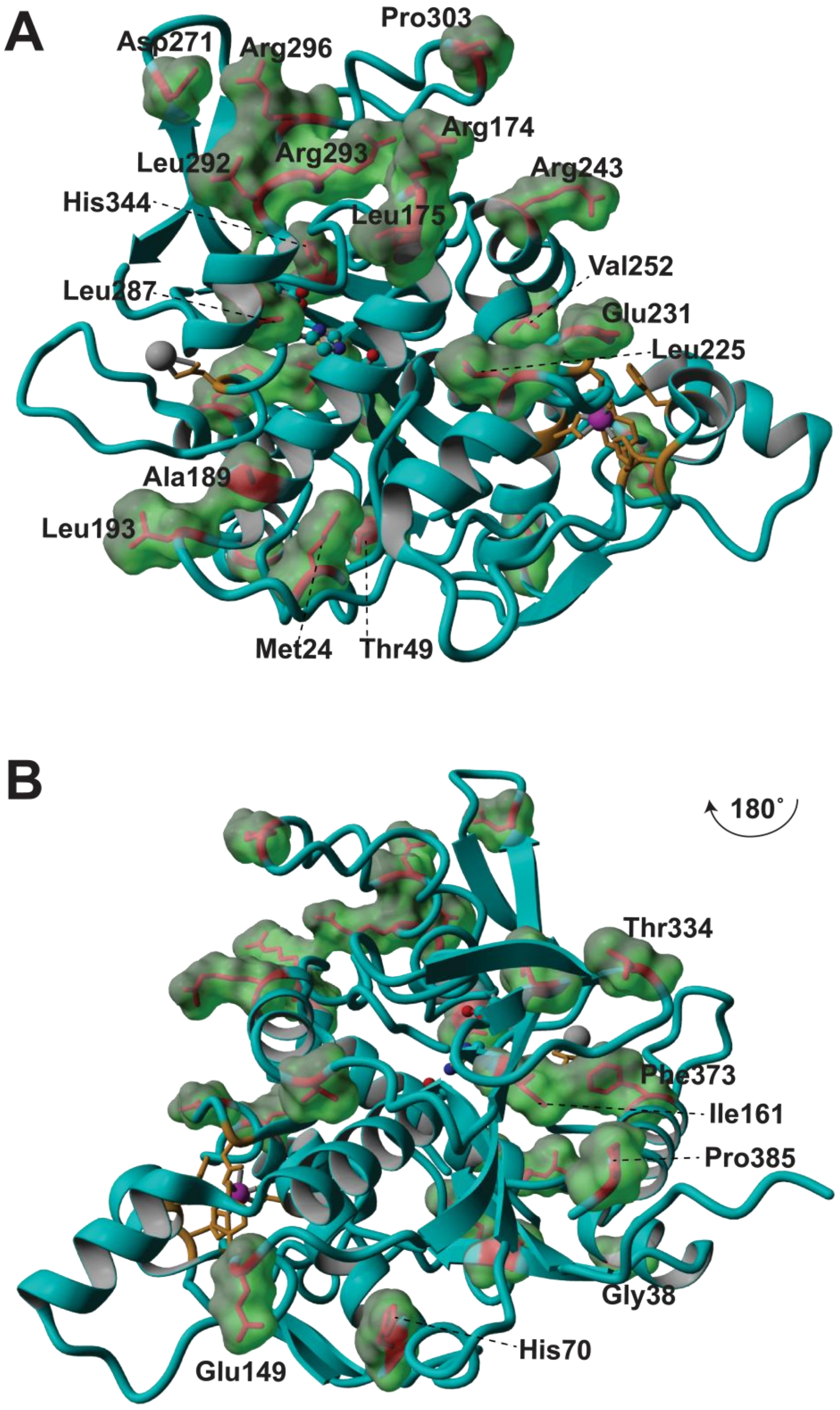

Supplementary Figure 4. (A) The modeled structure of NHS7108 displaying 24 amino acid substitutions, shown as red sticks and labeled in black. Residues interacting with Zn^{2+} (magenta sphere) and Ca^{2+} (grey sphere) metals are depicted in orange. (B) A 180º rotation of the structure is provided to enhance visualization of the amino acid substitutions at positions Gly38, His70, Glu149, Ile161, Thr334, Phe373, and Pro385.

Supplementary Table 1. Summary of the administration of various doses of NHS7108 and commercial PERT to PDL minipigs, along with the corresponding calculated %CFA values for each treatment group. Pre- and post-dose %CFA values were determined using data from the final count of animals per group as shown below, excluding minipigs with pre-dose %CFA above 60%. Data are represented as mean ± SEM. Δ is defined as ($\%CFA^{post\text{-}dose}$-$\%CFA^{pre\text{-}dose}$). (As outlined in the Methods section, purified NHS7108 was administered as lyophilized fermentation powder, while PERT was given in the form of enterically coated minitablets.

				**%CFA**			
**Treatment**	**Units/day (in 1000s)**	**Amount (g)**	**Final number of animals per group**	**Pre-surgery**	**Pre-dose**	**Post-dose**	**Δ**
NHS7108	33.7	0.055	2	93±1.5	40±4	65.5±2	25.5±6
	100	0.17	3		37.5±4	74±1	36.5±5
	301	0.5	3		42±6	81±1	39±7
PERT	80	1.4	4		50±7	72±3	22±10
	200	3.4	4		44±4	78±4	34±8
	400	6.8	4		44.5±4	84±2	39.5±6
